# CETSA quantitatively verifies *in vivo* target engagement of novel RIPK1 inhibitors in various biospecimens

**DOI:** 10.1038/s41598-017-12513-1

**Published:** 2017-10-12

**Authors:** Tsuyoshi Ishii, Takuro Okai, Misa Iwatani-Yoshihara, Manabu Mochizuki, Satoko Unno, Masako Kuno, Masato Yoshikawa, Sachio Shibata, Masanori Nakakariya, Takatoshi Yogo, Tomohiro Kawamoto

**Affiliations:** 1 Biomolecular Research Laboratories, Takeda Pharmaceutical Company Limited, 26-1, Muraoka-higashi 2-chome, Fujisawa, Kanagawa 251-8555 Japan; 2 Immunology Unit, Takeda Pharmaceutical Company Limited, 26-1, Muraoka-higashi 2-chome, Fujisawa, Kanagawa 251-8555 Japan; 3 Drug Metabolism & Pharmacokinetics Research Laboratories, Takeda Pharmaceutical Company Limited, 26-1, Muraoka-higashi 2-chome, Fujisawa, Kanagawa 251-8555 Japan

## Abstract

The proof of target engagement (TE) is a key element for evaluating potential investment in drug development. The cellular thermal shift assay (CETSA) is expected to facilitate direct measurement of intracellular TE at all stages of drug development. However, there have been no reports of applying this technology to comprehensive animal and clinical studies. This report demonstrates that CETSA can not only quantitatively evaluate the drug-TE in mouse peripheral blood, but also confirm TE in animal tissues exemplified by using the receptor interacting protein 1 kinase (RIPK1) lead compound we have developed. Our established semi-automated system allows evaluation of the structure-activity relationship using native RIPK1 in culture cell lines, and also enables estimation of drug occupancy ratio in mouse peripheral blood mononuclear cells. Moreover, optimized tissue homogenisation enables monitoring of the *in vivo* drug-TE in spleen and brain. Our results indicate that CETSA methodology will provide an efficient tool for preclinical and clinical drug development.

## Introduction

A large number of drug candidates have failed in clinical trials because of not only lack of efficacy but also non-verification of the predicted pharmacological mechanism of action due to insufficient interpretation of fundamental pharmacokinetic/pharmacodynamic principles, target engagement (TE), and expression of functional pharmacological activity^1,2^. TE is one of the key elements to reduce the high failure rates in clinical trials^3^. Therefore, robustness of the measurements of drug TE from the initial stage of drug discovery through to clinical development can provide a breakthrough for drug development.

The cellular thermal shift assay (CETSA) has recently been reported to monitor the binding of ligand to its target protein in cells and tissue samples. This method is based on the ligand-induced changes in protein thermal stability^4–6^. In pre-clinical and clinical stages, there are several kinds of TE assays, including prediction of potency based on compound concentration in tissue^7^, use of tracer molecules such as positron emission tomography (PET)^8,9^, and detection of substrate in the target compartment^7^. Compared with existing methods, CETSA has the capability to evaluate biophysical binding under physiological and pathological conditions without any special experimental tools. Therefore, this technology is expected to be applied to many stages of drug development. During the initial stages of CETSA application, much work has focused on TE experiments in cultured cells and verified the applicability to a variety of target families. However, there are only a few reports evaluating CETSA technology in animal and clinical studies. In the first of these, Molina *et al*. demonstrated *in vivo* TE with TNP-470 which is a covalent inhibitor against methionine aminopeptidase-2^6^. Another group demonstrated qualitative TE in a xenograft model using Michael acceptor inhibitor^10^. However, covalent drugs are rarely considered in target-directed drug discovery owing to safety concerns^11^. With regards to TE of a non-covalent compound using intact tissues, one group applied this technology to investigate histone deacetylase isoform selectivity of a compound with human brain homogenate^12^. Under these situations, one of the present challenges for CETSA technology is to quantitatively demonstrate TE in *in vivo* tissue with non-covalent compounds. To achieve this goal, maintaining compound concentrations *in vivo* is a key factor because reversible compounds leave the target protein when the concentration is less than the binding affinity between the compound and the target through the sample preparation processes. Therefore, it is necessary for the performance of challenges to establish the procedures for both tissue excision and sample preparation until the transient heating step.

Receptor interacting protein 1 kinase (RIPK1) is a key mediator of not only a process of regulated necrosis, termed necroptosis, but also promotion of caspase-8-dependent apoptosis and pro-inflammatory gene expression^13^. Based on kinase-dead knock-in RIPK1 mice and highly selective allosteric Type 3 RIPK1 inhibitors (necrostatin-1 [Nec-1] and optimized analogue Nec-1s)^14,15^, RIPK1 is implicated in a variety of human diseases, such as ischemia-reperfusion injury in the brain^16^, heart^17^, and kidney^18^, acute and chronic inflammatory diseases^19^, multiple sclerosis (MS)^20^, and amyotrophic lateral sclerosis^21^. Recently, our group has developed a reversible, highly potent lead compound **22**, with high kinase-selectivity and excellent pharmacokinetics^22^. After oral administration of this compound to mice, the unbound concentrations in spleen and brain are sufficient to show *in vivo* inhibition of mouse endogenous RIPK1. In fact, this compound exhibits activity in an experimental autoimmune encephalomyelitis (EAE) model^22^, which is the most commonly used experimental model for MS^23^. Since MS is the prototypical inflammatory demyelinating disease of the central nervous system, these results suggest that compound **22** might bind the endogenous RIPK1 in brain tissue in order to exhibit pharmacological activity. What is particularly interesting is the TE of this compound **22** in the animal brain.

Here, we demonstrate that CETSA is feasible for evaluating the TE of reversible kinase inhibitors in *in vivo* animal experiments exemplified by our recently developed RIPK1 inhibitors. To our knowledge, there has been no report to demonstrate TE for reversible inhibitors in animal experiments. Using an established semi-automated system, the drug occupancy ratio in peripheral blood mononuclear cells (PBMCs) is estimated, and direct binding of RIPK1 inhibitor on *in vivo* RIPK1 is successfully monitored in brain and spleen samples. Therefore, the use of both appropriately-prepared both PBMCs and tissue biopsy samples for TE could be as a biomarker in future clinical trials. Our study verifies that CETSA could serve as a powerful tool for animal and clinical studies.

## Results

### Semi-automated CETSA evaluating TE in cells

**Figure 1:**
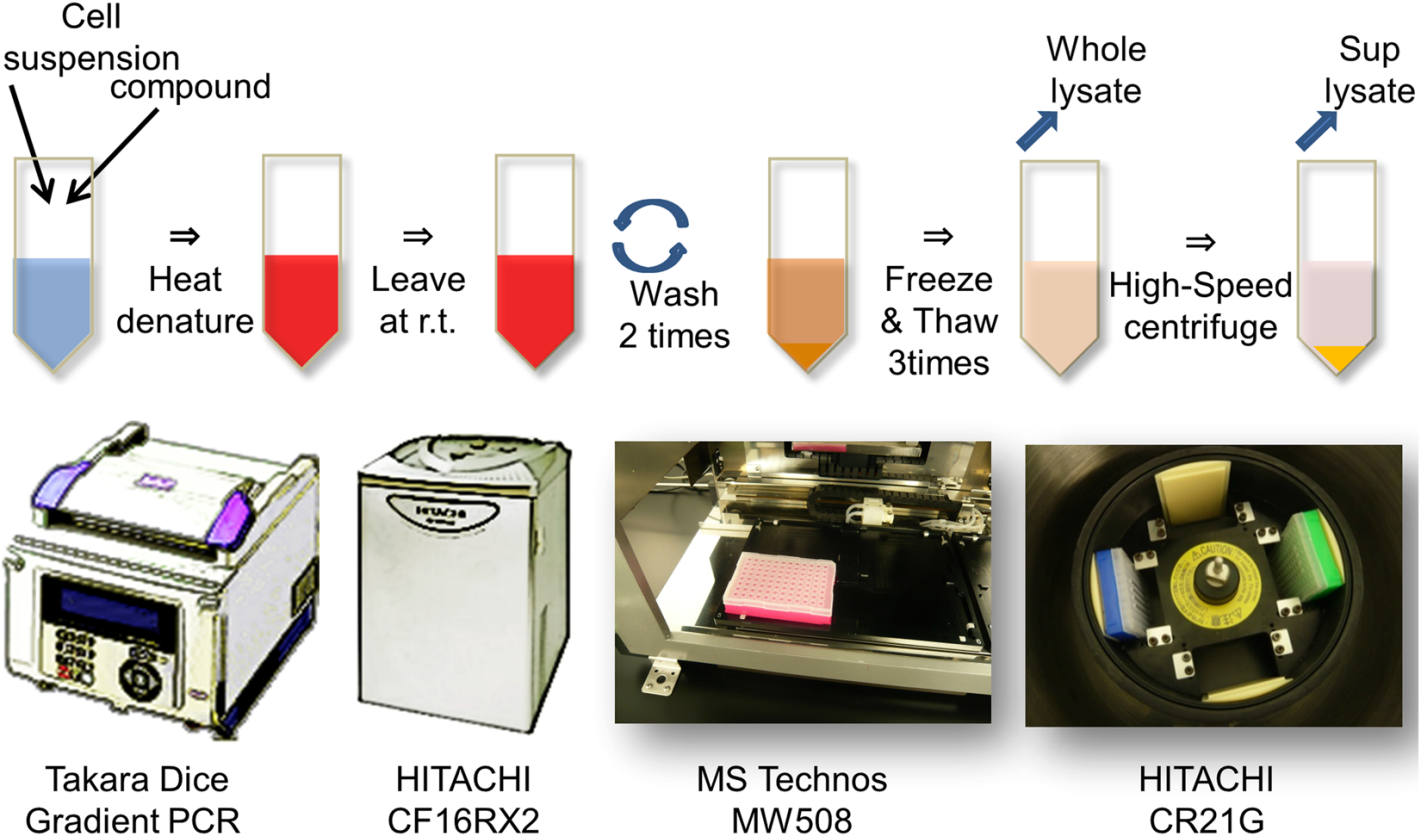
Development of semi-automated system of CETSA wash in 96-well plates. Illustration of semi-automated system of CETSA for both culture cells and PBMCs is described. Both T_agg_ curve and ITDR experiments are performed with Gradient PCR. After heat denature, culture medium and plasma in samples are removed and cells are washed with an automated pipetting and dispensing system. A CR21G high-speed refrigerated centrifuge was used to centrifuge the 96-well plates. Details are given in Methods. CETSA, cellular thermal shift assay; ITDR, isothermal dose-response; PBMC, peripheral blood mononuclear cell; PCR, polymerase chain reaction; T_agg_, aggregation temperature.

To establish this efficient and feasible detection method, we first tried to develop a sandwich enzyme-linked immunosorbent assay (ELISA) to monitor the quantity of RIPK1 protein. However, compound-induced quenching of protein target recognition was observed by both our ELISA assay using several combinations of RIPK1 antibodies and a commercially available ELISA kit. This quenching effect has already been reported^4,24^ and the effects on RIPK1 protein might be dependent on RIPK1 associated with a variety of proteins^14,15,25^. Therefore, we focused on establishing a widely used Western blotting method in a semi-automated procedure. The semi-automated system used both an automated pipetting and dispensing system and a 96-well high-speed refrigerated centrifuge. The principle of CETSA technology is to detect the remaining soluble target protein from a background of thermally denatured and precipitated proteins after heat denaturation^5,26^. The washing process to remove unwanted components from samples is important to reduce the background noise of detection systems, because both animal plasma and cell culture media have high protein concentrations. As outlined in Fig. 1, either culture cells or PBMCs are first treated with compounds in 96-well polymerase chain reaction (PCR) plates. The compound-treated samples are transiently heated in a Takara Dice Gradient PCR (TAKARA), which is able to control a series of different temperatures in one 96-well PCR plate. After heating the intact cells, washing is carried out with a combination of low-speed centrifuge and MW508 liquid handling machines for the 96-well PCR plates. Next, three freeze-thaw cycles using liquid nitrogen are performed on the heat-treated cell suspensions. Finally, a 96-well high-speed refrigerated centrifuge efficiently separates the remaining soluble target protein in the supernatant from the thermally denatured and precipitated proteins. Even though the intact cells were washed, compound-induced quenching of protein target recognition was observed by our ELISA assay. Therefore, a widely used Western blotting method in a semi-automated procedure was essential for our evaluations.

We validated our semi-automated procedure with a trypan blue exclusion test and actual CETSA experiments. To optimize the liquid handling systems, the effluent of the wash process was monitored with a trypan blue exclusion test and no cells were detectable in the effluent. By our calculations, this wash process can provide an almost 200-fold dilution of the protein concentration in cell culture media. Using this automation system, all the experiments for both culture cells and PBMCs were performed in duplicate wells, and the small variations indicated adequate quality for CETSA. Isothermal dose-response fingerprint (ITDRF) experiments in human colorectal adenocarcinoma HT-29 cells demonstrated high reproducibility for two compounds in independent experiments (Supplementary Fig. 1a,b).

### Development of CETSA for human RIPK1

**Figure 2:**
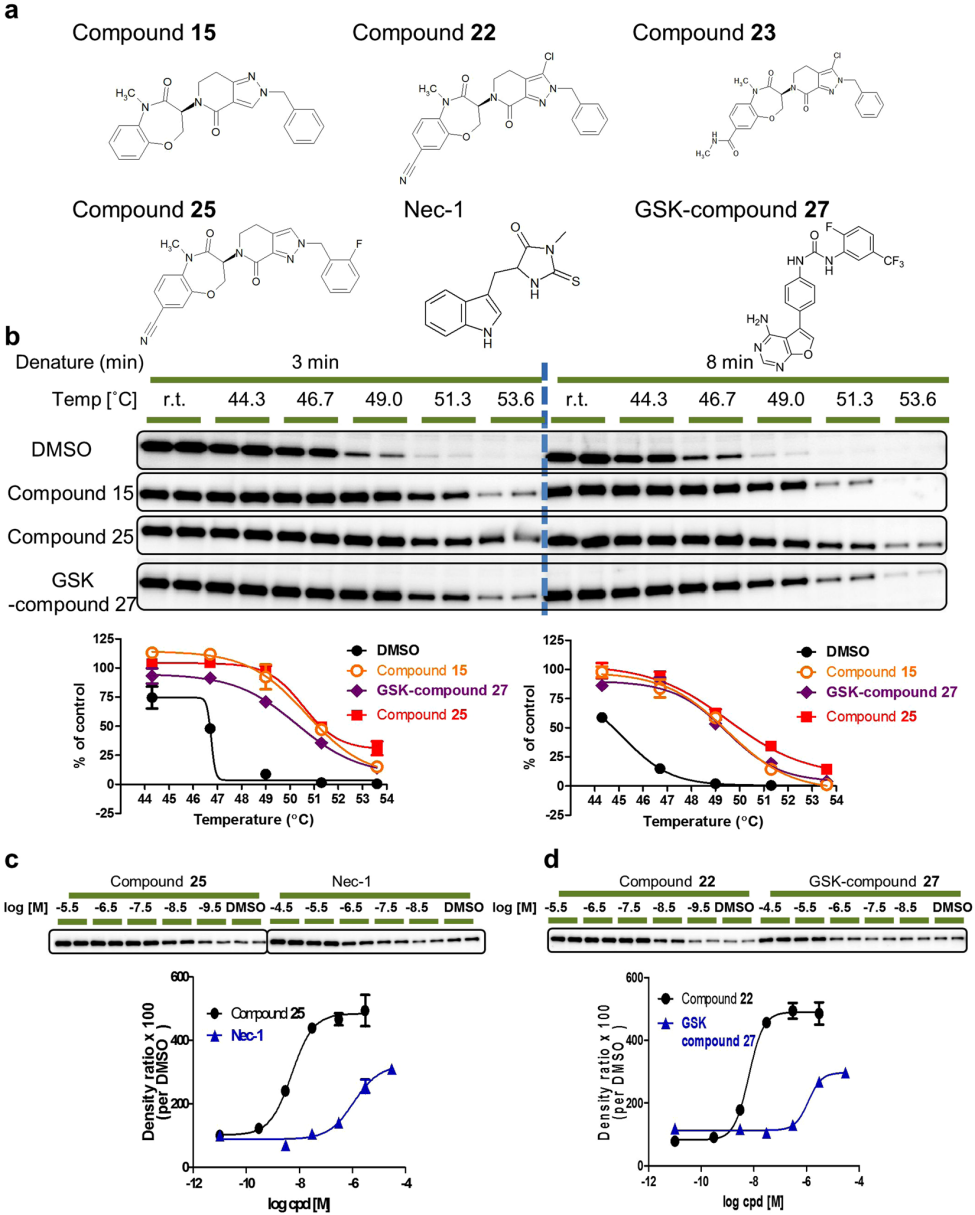
Development of a CETSA for human RIPK1 with HT-29 cells. (**a**) Chemical structure of representative tool compounds. (**b**) T_agg_ curves for RIPK1 in HT-29 cells in the presence of DMSO (0.1%) (black closed circle), 10 μM compound **25** (red square), GSK-compound **27** (purple diamond), or compound **15** (orange open circle). Evaluation of 3 min and 8 min denature conditions was performed to verify the optimized conditions. All data were normalised to the response observed at DMSO-treated conditions at room temperature. The T_agg_ shift was analysed using the Boltzmann sigmoid equation. The vertical dotted line is at 47 °C for 8 min denature, the experimental condition selected for the ITDRF assay. Data are provided as the average and SEM performed in duplicate. (**c,d**) ITDRF of representative RIPK1 inhibitors at 47 °C for 8 min denature based on raw data from the Western blotting chemiluminescence readings. The chemiluminescence data are shown above the graphs. ITDRF lines are fitted with a four-parameter logistic curve. The corresponding ITDRF for compound **25** (**c**, black circle), Nec-1 (**c**, blue triangle), compound **22** (**d**, black circle), and GSK-compound **27** (**d**, blue triangle) result in EC_50_ of 5.0 nM (95% CI 2.8–9.1), 1,100 nM (95% CI 500–2,400), 6.5 nM (95% CI 4.3–9.8), and 1,200 nM (95% CI 810–1,700), respectively. Data are provided as the average and SEM performed in duplicate. All full-length Western blotting images are presented in Supplementary Fig. 8. CETSA, cellular thermal shift assay; DMSO, dimethyl sulfoxide; ITDRF, isothermal dose-response fingerprint; RIPK1, receptor interacting protein 1 kinase; SEM, standard error of the mean; T_agg_, aggregation temperature.

In order to confirm the feasibility of CETSA for human RIPK1, we established an ITDRF assay with HT-29 cells using the semi-automated system described earlier. Several types of RIPK1 inhibitors have been reported^16,22,27–30^. We validated our assay by investigating the dose response curves of a total of 14 compounds: Nec-1, GSK-compound **27**, and twelve 7-oxo-2,4,5,7-tetrahydro-6H-pyrazolo[3,4-c]pyridine derivatives (Fig. 2a, Supplementary Table 1). Firstly, aggregation temperature (T_agg_) curves were analysed at a series of different temperatures with 3 or 8 min denaturation at 10 μM fixed dose for three compounds, compound **15**, compound **25**, and GSK-compound **27** (Fig. 2b). All three compounds showed substantial shifts of the thermal stability of RIPK1 under the two different denaturation times. An 8 min denaturation time results in lower apparent T_agg_ in comparison with a 3 min denaturation. Over all temperature conditions, the integrity of the cell membrane in HT-29 cells was confirmed with a trypan blue dye exclusion experiment (Supplementary Fig. 2a). Moreover, the total protein of RIPK1 was not changed with treatment of three compounds at room temperature, even in the presence of 10 μM (Fig. 2b). Based on these T_agg_ curves, denaturation at 47 °C for 8 min was selected for the next evaluation of ITDRF experiments. HT-29 cells were treated with serially titrated compounds for 30 min, after which all samples were heated. Four compounds showed dose-dependent stabilisation of RIPK1 with different half-maximal effective concentration (EC_50_) values (Fig. 2c,d). The EC_50_ values were calculated with duplicate data points and the standard error of the mean (SEM) demonstrated small variations. In order to evaluate repeatability of this ITDRF assay, the reproducibility of the EC_50_ evaluation was performed in multiple experimental runs for two compounds. The ITDRF of compound **25** exhibited an EC_50_ of 4.9 nM (95% confidence interval [CI] 1.0–24) and 5.0 nM (95% CI 2.8–9.1) (Supplementary Fig. 1a) and GSK-compound **27** resulted in an EC_50_ of 1,100 nM (95% CI 700–1,700), 640 nM (95% CI 350–1,200), and 1,200 nM (95% CI 810–1,700) (Supplementary Fig. 1b). These results suggested that the ITDRF for human RIPK1 in HT-29 cells is a feasible and robust assay. ITDRF-CETSA methodology depends on the irreversible aggregation of denatured proteins. Our curve fitting formula makes use of equilibrium models for data analysis Therefore, it is not appropriate to estimate the real binding affinities of the compound to intercellular RIPK1. However, there are no consensus formulas. For this reason, we refer to the observed responses as apparent binding affinity.

**Figure 3:**
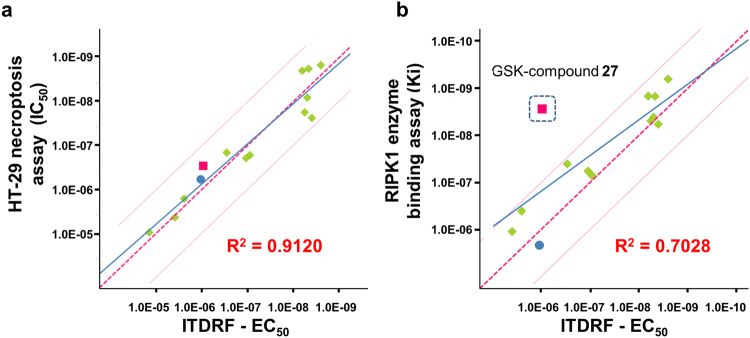
Correlation with both necroptosis functional assay and RIPK1 binding assay. (**a**) Scatter plots of IC_50_ of HT-29 necroptosis inhibitory activities with ITDRF values of half-maximal stabilization of endogenous RIPK1 for 14 representative RIPK1 inhibitors. Dotted red line presents Y = X and solid blue line indicates the regression curve. Decision coefficient R^2^ for all data points is 0.9120. (**b**) Scatter plots of IC_50_ of recombinant RIPK1 binding assay with ITDRF values of half-maximal stabilization of endogenous RIPK1 for 14 representative RIPK1 inhibitors. Dotted red line presents Y = X and solid blue line indicates the regression curve. Decision coefficient R^2^ for all data points is 0.7028. IC_50_, half-maximal inhibitory concentration; ITDRF, isothermal dose-response fingerprint; RIPK1, receptor interacting protein 1 kinase.

In order to evaluate the relationship among biophysical binding affinities under both intracellular and *in vitro* recombinant enzyme conditions, and functional cellular activity, further evaluations were performed with 14 RIPK1 compounds (Fig. 3a,b). The efficacies of RIPK1 compounds were tested in *in vitro* necroptosis assay using HT-29 cells, which were treated with tumour necrosis factor alpha (TNF-α) in presence of the Smac mimetic birinapant and the pan-caspase inhibitor z-VAD-FMK^31^. The binding affinities of compounds on recombinant RIPK1 proteins were monitored through fluorescence resonance energy transfer (FRET) competitive binding assay. The values of ITDRF are almost similar to that of the half-maximal inhibitory concentration (IC_50_) in the necroptosis assay. Significant positive linear correlation between ITDRF and necroptosis assay was demonstrated (decision coefficient: R^2^ = 0.9120) (Fig. 3a). Moreover, there was a positive correlation for the ITDRF and RIPK1 recombinant enzyme assay (decision coefficient: R^2^ = 0.7028) (Fig. 3b). The lower values for R^2^ in enzyme-binding assay are highly dependent on GSK-compound **27** having higher potent affinity for recombinant RIPK1 compared with ITDRF assay. Except for GSK-compound **27**, the values of ITDRF show almost the same IC_50_ values in the RIPK1 enzyme-binding assay. Even though ITDRF is based on the irreversible precipitation of unfolded proteins and our fitting formula is not sufficient for the real binding affinities within cells, the positive correlation among ITDRF, necroptosis assay, and RIPK1 recombinant enzyme assay suggested that ITDRF-CETSA might provide the quantitative interpretation in the case of RIPK1 under our experimental conditions. These results suggested that ITDRF assay in HT-29 cells may allow us to evaluate the intracellular structure-activity relationship (SAR) for RIPK1 in a native cellular environment.

### Development of CETSA for mouse RIPK1

Before applying the CETSA method to *in vivo* experiments, the feasibility of CETSA for mouse RIPK1 was evaluated with mouse L-cells NCTC 929 (L929). The assay development was conducted in the similar way to that of the human RIPK1 CETSA assay. Up to 60 °C, the integrity of the cell membrane of L929 cells was confirmed by trypan blue dye exclusion, even with 10 μM representative RIPK1 inhibitors (Supplementary Fig. 2b). T_agg_ curves were analysed with 3 or 8 min denaturation at a series of different temperatures at 10 μM fixed dose of compound **25** (Supplementary Fig. 3a–c). In the same way as T_agg_ curves in human RIPK1, compound **25** showed substantial shifts of the thermal stability of mouse RIPK1 under the two different denaturation times. Based on these T_agg_ curves, the experimental condition for mouse RIPK1 was set at the same experimental conditions as for human RIPK1 for the next evaluation of ITDRF experiments, i.e. denaturation at 47 °C for 8 min. The derivatives of 7-oxo-2,4,5,7-tetrahydro-6H-pyrazolo[3,4-c]pyridine have much lower inhibitory activity on mouse RIPK1 than human RIPK1 in recombinant enzyme assays (Supplementary Table 1). Therefore, a representative six compounds were analysed with the ITDRF assay in L929 cells. L929 cells were also treated with serially titrated compounds for 30 min, after which all samples were heated. These six compounds also exhibited dose-dependent stabilization of mouse RIPK1 with different EC_50_ values (Supplementary Fig. 3d–f). The EC_50_ values for 7-oxo-2,4,5,7-tetrahydro-6H-pyrazolo[3,4-c]pyridine derivatives and Nec-1 showed almost similar Ki values to recombinant mouse RIPK1, although GSK-compound **27** showed an almost 500-fold lower Ki value in comparison with the EC_50_ in mouse ITDRF assay (Supplementary Table 1). This result is correlated with the assay results of ITDRF assay for human RIPK1. ITDRF for mouse RIPK1 might also suggest the quantitative interpretation under our experimental conditions.

In order to evaluate the relationship between intracellular biophysical binding affinity and functional cellular activity, further evaluations were performed with two types of cellular assays, necroptosis assay and phospho (Ser-345)- Mixed lineage kinase domain-like protein (MLKL) ELISA assay. The efficacies of RIPK1 compounds were tested *in vitro* necroptosis assay using L929 cells, which were treated with necroptosis inducer containing mouse TNF-α and Z-VAD-FMK^31^. RIPK1 regulates RIPK3-MLKL-driven necroptosis and RIPK3 phosphorylates MLKL at Ser345^25,32^. Therefore, we developed the phospho (Ser-345)-MLKL ELISA assay as a proximal pharmacodynamics marker and the effects of inhibitors on this event were evaluated with representative RIPK1 compounds. All three 7-oxo-2,4,5,7-tetrahydro-6H-pyrazolo[3,4-c]pyridine derivatives (compound **22, 23**, and **25**) showed inhibitory activities on cellular functional assays (Table 1). The high correlation between L929 necroptosis assay and p-MLKL assay were observed (Table 1). The differences in IC_50_ values for each compound were less than 2.5-fold. Moreover, the order of inhibitory activities on recombinant enzyme assay also correlated with two types of cellular functional assays, even though IC_50_ values in cellular assays were almost 10-fold lower than Ki values with mouse recombinant enzyme (Table 1). These results suggested that ITDRF assay in mouse L929 cells can also be used to evaluate the SAR for mouse RIPK1 in a native cellular environment.

**Table 1:** Tool inhibitors in mouse assay.

	Compound 22	Compound 23	Compound 25
hRIPK1 enzyme (Ki)	1.5 nM (1.3 nM–1.6 nM)	1.5 nM (1.4 nM–1.6 nM)	4.1 nM (3.8 nM–4.4 nM)
mRIPK1 enzyme (Ki)	140 nM (120 nM–160 nM)	130 nM (110 nM–150 nM)	840 nM (720 nM–970 nM)
L929-necroptosis (IC_50_)	15 nM (14 nM–18 nM)	15 nM (14 nM–18 nM)	140 nM (140 nM–150 nM)
L929-pMLKL (IC_50_)	12 nM (11 nM–13 nM)	16 nM (11 nM–23 nM)	66 nM (55 nM–80 nM)
L929-CETSA (EC_50_)	120 nM (91 nM–160 nM)	290 nM (250 nM–340 nM)	1,600 nM (1,200 nM–2,100 nM)

CETSA, cellular thermal shift assay; EC_50_, half-maximal effective concentration; IC_50_, half-maximal inhibitory concentration; Ki, inhibitory constant; RIPK1, receptor interacting protein 1 kinase.

### *Ex vivo* mouse PBMC evaluation

**Figure 4:**
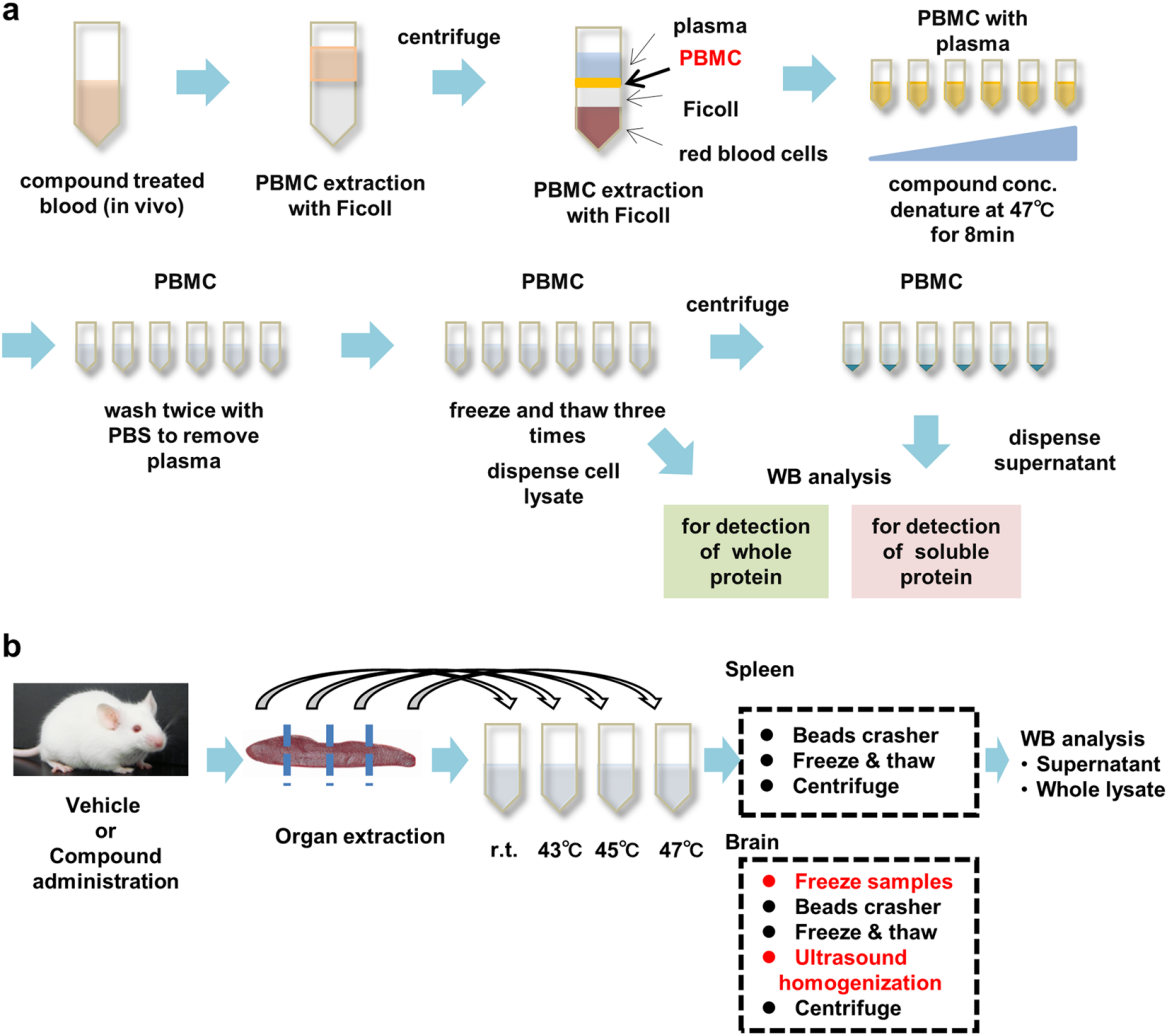
Experimental procedures for *in vivo* and *ex vivo* CETSA. Overview of CETSA sample preparation for both PBMC and tissues (spleen and brain) is described. (**a**) *In vivo* and *ex vivo* PBMC sample preparation. For *in vivo* evaluation, compounds are administered to C57BL/6 J mice. After the prescribed time, mouse peripheral blood is collected and PBMCs are isolated by density centrifugation over Ficoll according to the manufacturer’s instructions. For *ex vivo* evaluation, after the isolation of PBMCs with the Ficoll density centrifugation method, PBMCs are mixed with each compound. The isolated PBMC solutions are divided into 96-well PCR plates. The procedures for sample heat treatment and washing are described in Fig. 1. Both whole protein and supernatant samples are reserved for Western blot analysis. (**b**) *In vivo* spleen and brain sample preparation. Dissected spleen and brain samples are divided into four approximately equal parts. The samples are added to pre-warmed PBS containing protease inhibitor and incubated for 8 min at regulated temperatures. In the case of brain, the heat-treated samples are frozen using liquid nitrogen, and then homogenised with bead beaters. After bead homogenisation, the samples are freeze-thawed three times, and then homogenised by ultrasonic tissue homogenisation. For the spleen, preparation of the samples excluded initial sample freezing and ultrasonic tissue homogenisation. In the same way as PBMC sample preparation, both whole protein and supernatant samples are reserved for Western blot analysis. CETSA, cellular thermal shift assay; PBMC, peripheral blood mononuclear cell; PCR, polymerase chain reaction; WB, western blot.

To expand the CETSA application to comprehensive animal studies, we first selected the use of PBMCs for future preclinical and clinical drug development. Whole blood contains ~45% by volume of red cells, and these cells contain haemoglobin, a complex protein containing iron that carries oxygen through the body. To reduce the influence of the abundant protein derived from red cells, we used PBMCs in blood. In order to monitor the TE of mouse RIPK1 in PBMCs, PBMCs were isolated by density gradient media of Ficoll-fluid. The PBMCs were treated with serially titrated compounds for 30 min, and were then heat-denatured at 47 °C for 8 min. To improve accuracy, both whole protein and the remaining soluble target protein samples were analysed for Western blot analysis (Fig. 4a) and the thermal stabilities were estimated supernatant per whole ratios for heat-denatured samples.

**Figure 5:**
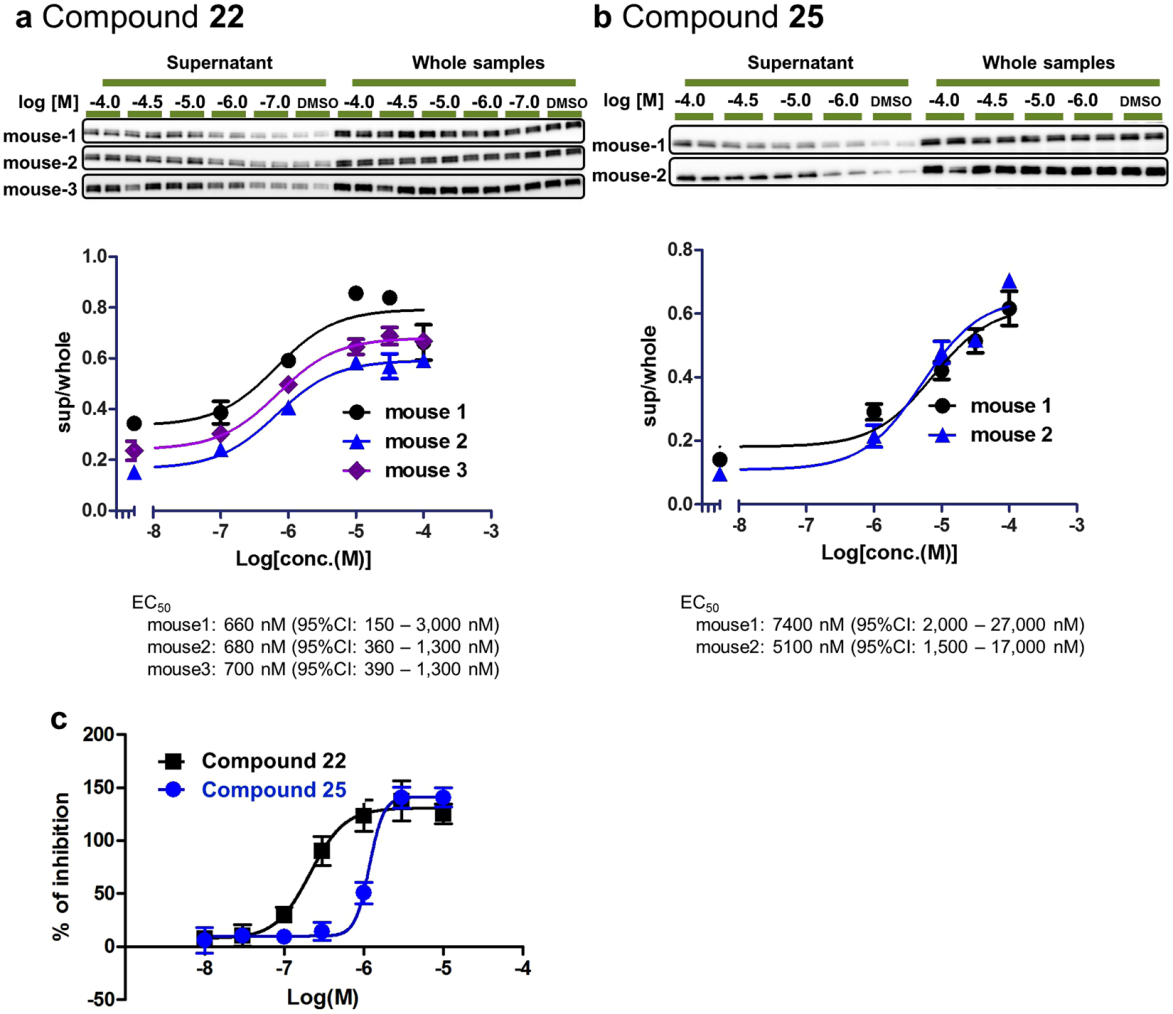
*Ex vivo* PBMC assays. (**a**,**b**) ITDRF EC_50_ evaluation for compound 22 and compound 25 in PBMCs. Using aliquots of isolated PBMCs suspended in plasma, the compounds were mixed with the PBMCs for 30 min. The cell samples were heat treated at 47 °C for 8 min. The experimental protocol is described in Methods. ITDRF EC_50_ evaluation of compound **22** was performed for PBMCs derived from three individual mouse plasma samples and that of compound **25** was evaluated with two individual PBMC samples. (**a**) The corresponding ITDRFs of compound **22** for mouse 001 (black circle), mouse 002 (blue triangle), and mouse 003 (purple diamond) resulted in EC_50_ of 660 nM (95% CI 150–3,000), 680 nM (95% CI 360–1,300), and 700 nM (95% CI 390–1,300), respectively. (**b**) The corresponding ITDRFs of compound **25** for mouse 001 (black circle) and mouse 002 (blue triangle) resulted in EC_50_ of 7,400 nM (95% CI 2,000–27,000) and 5,100 nM (95% CI 1,500–17,000), respectively. The chemiluminescence intensities of supernatant data were normalised to the response observed at the intensities of corresponding whole protein data. Data are provided as the average and SEM performed in duplicate. All full-length Western blotting images are presented in Supplementary Fig. 9. (**c**) Dose response curves for compound **22** and compound **25** in the *in vitro* whole blood necroptosis assay. The IC_50_ values of compound **22** (black square) and compound **25** (blue circle) resulted in 210 nM (95% CI 160–280) and 1,200 nM (95% CI 840–1,700), respectively. Data are provided as the average and SEM performed in triplicate. CI, confidence interval; EC_50_, half-maximal effective concentration; IC_50_, half-maximal inhibitory concentration; ITDRF, isothermal dose-response fingerprint; PBMC, peripheral blood mononuclear cell; SEM, standard error of the mean.

ITDRF EC_50_ valuation of compound **22** was performed for PBMCs derived from three separate mouse plasma samples and that of compound **25** was evaluated with two separate mice-derived PBMC samples (Fig. 5a,b) The corresponding ITDRFs of compound **22** for three mice resulted in EC_50_ of 660 nM (95% CI 150–3,000), 680 nM (95% CI 360–1,300), and 700 nM (95% CI 390–1,300). The ITDRFs of compound **25** for two mice resulted in EC_50_ of 7,400 nM (95% CI 2,000–27,000) and 5,100 nM (95% CI 1,500–17,000). There seemed to be not much differences between PBMCs derived from different mice. In comparison with ITDRF EC_50_ values in L929 cells, the EC_50_ values in mouse PBMCs exhibited almost 5-fold lower values, but the order of the affinities in PBMCs correlated with those in L929 cells (Fig. 5a,b, Table 1). Unbound fractions in plasma of compound **22** and compound **25** were 0.060 and 0.13, respectively (Supplementary Table 2). The culture media of L929 contains 10% foetal bovine serum (FBS), but PBMCs were cultured in 100% plasma. With the correction for dilution factor according to the Kalvass and Maurer proposed equation^33^, unbound fractions of compound **22** and compound **25** in 10% plasma were 0.39 and 0.60, respectively. Even though L929 cells were cultured in 10% FBS, the almost 5-fold difference in EC_50_ values might be due to the unbound fraction of the compounds. In another aspect, the thermal stability of intracellular RIPK1 might be due to the type of cells, although heat-denaturing condition was same for both PBMCs and L929 cells. Moreover, no change of RIPK1 protein in whole samples was observable at all compound concentration ranges in PBMCs, demonstrating that the thermal stabilization data were not influenced by drug-induced change in total protein level.

To evaluate the relationship between ITDRF values and functional cellular activity in PBMCs, further evaluations were performed with whole blood necroptosis assay. This assay monitors the inhibition of monocyte cell death when whole mouse blood is treated with both RIPK1 compound and necroptosis inducers (Supplementary Figs 4 and 5). The IC_50_ values of compound **22** and compound **25** were 210 nM (95% CI 160–280) and 1,200 nM (95% CI 840–1,700), respectively (Fig. 5c). The ITDRF values for the two compounds in PBMCs were in the same order as the IC_50_ values in whole blood assay. These data suggested that a combination of CETSA and PBMCs is a promising technology to evaluate TE in animal blood.

### *In vivo* mouse PBMC evaluation

Since mouse RIPK1 ITDRF assay is applicable using *ex vivo* PBMCs, we applied this methodology to RIPK1 occupancy analysis for compound **22** in the oral dosing of mice. Compound **22** is a highly potent, highly kinase selective lead compound with excellent pharmacokinetic qualities. Furthermore, this compound has shown activity in an EAE model and pharmacokinetic studies have demonstrated the brain permeability of the compound^22^. With respect to cellular thermal stability, this compound increased only the thermal stability of intracellular RIPK1, but not RIPK2, RIPK3, or β-actin using HT-29 cells (Supplementary Fig. 6a).

**Figure 6:**
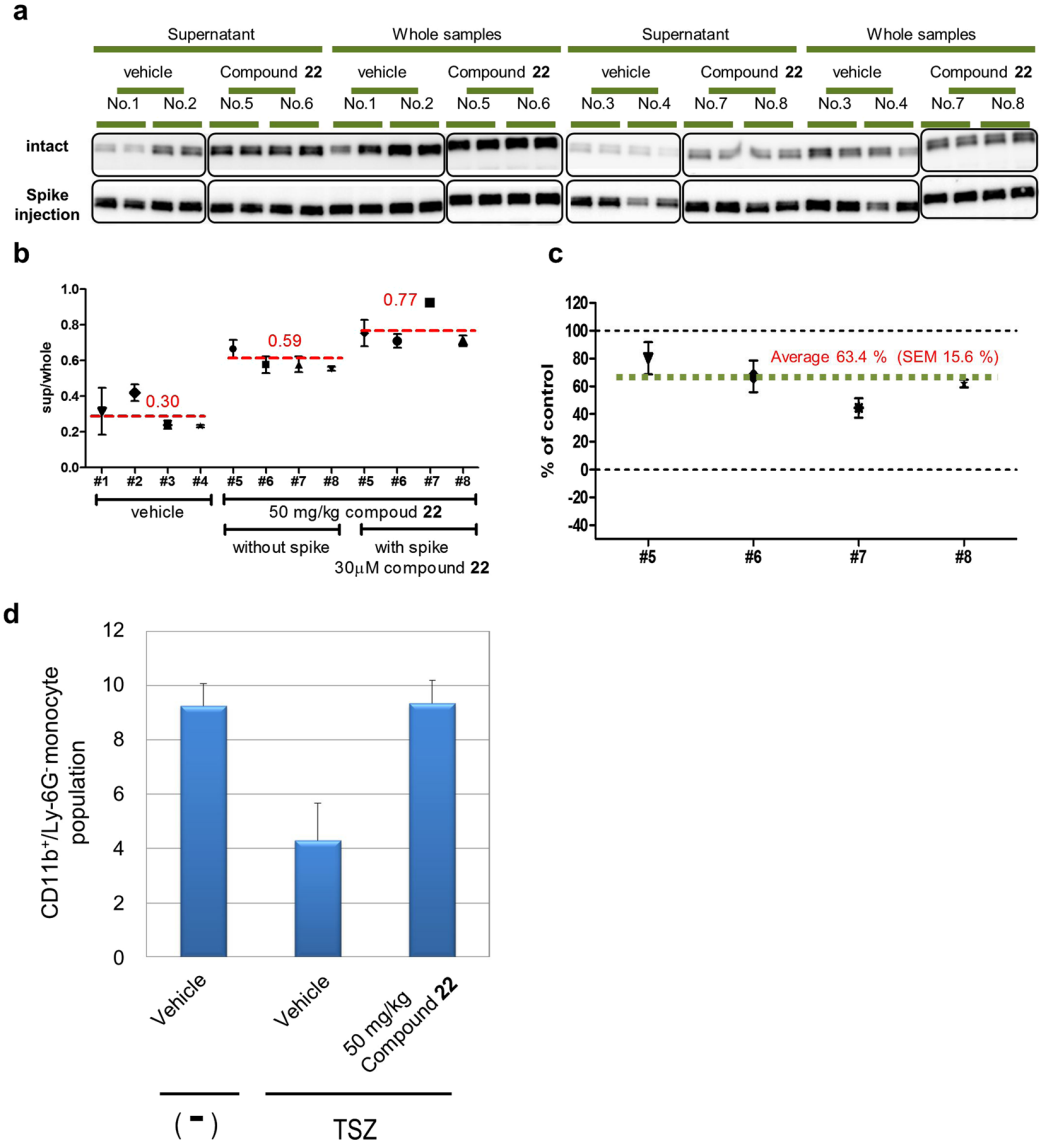
*In vivo* PBMC CETSA. Isothermal experiments for PBMCs from mice with orally administered compound **22**. Mice were divided into two groups of four mice each, vehicle control and experimental mice, orally dosed with 50 mg/kg compound **22**. One hour after drug administration, mice were euthanized and blood was collected. (**a**) Western blot analysis for isolated PBMCs and compound **22** spike-injected samples. To determine the maximum occupation of RIPK1 in PBMCs, a portion of the isolated PBMCs was incubated with 30 μM compound **22** at 37 °C for 30 min for each sample. Analysis of each sample was performed in duplicate. All full-length Western blotting images are presented in Supplementary Fig. 10. (**b**) Thermal stability was estimated from supernatant per whole ratio. The chemiluminescence intensities of supernatant data were normalised to the response observed at the intensities of corresponding whole protein data. Data are provided as the average and SEM performed in duplicate. (**c**) Occupancy analysis for PBMCs from mice orally administered with compound **22**. Occupancy rate for each animal is calculated from supernatant per whole ratio by using the following formula; % of control = (X − B) × 100/(A − B); A, 30 μM compound **22** spike-injected sample; B, average of vehicle controls; X, compound-administered sample. (**d**) Whole blood necroptosis assay. Peripheral blood was collected from mice 1 h after oral administration of 50 mg/kg compound **22** and applied for whole blood necroptosis assay. Then CD11b + Ly-6G- monocyte population was calculated from flow cytometry analysis. Data are provided as the average and SEM performed in quadruplicate. CETSA, cellular thermal shift assay; PBMC, peripheral blood mononuclear cell; RIPK1, receptor interacting protein 1 kinase; SEM, standard error of the mean.

Based on the *ex vivo* results (Fig. 5a–c), the EC_50_ values of compound **22** in the ITDRF assay were almost the same as for the bloods derived from three different mice, but the B_max_ of supernatant per whole ratios were slightly different for each animal’s peripheral blood. To calculate the occupancy of native RIPK1 by compound **22**, a part of the isolated PBMCs was incubated with 30 μM compound **22** for 30 min at 37 °C for each sample, at which concentration the RIPK1 in the PBMCs should be completely occupied. Mice were divided into two groups of four animals each, vehicle control and experimental mice, orally dosed with 50 mg/kg compound **22**. One hour after drug administration, the mice were euthanized and blood was collected. Western blot analysis was performed for isolated PBMCs and compound **22** spike-injected samples in the same way as for *ex vivo* PBMC evaluation (Fig. 6a). The averages of the supernatant per whole ratios for vehicle, 50 mg/kg compound **22**, and 50 mg/kg compound **22** plus 30 μM compound **22** spike-injected samples, were 0.30, 0.59, and 0.77, respectively (Fig. 6b). Occupancy rate for each animal is calculated from supernatant per whole by using the following formula: % of control = (X − B) × 100/(A − B); A, 30 μM compound **22** spike-injected sample; B, average of vehicle controls; X, compound-administered sample. The average occupancy was 63.4% of endogenous RIPK1 in PBMCs. An *ex vivo* PBMC ITDRF assay demonstrated that average of EC_50_ value for compound **22** was 680 nM (Fig. 5a). Therefore, 63.4% occupation of intracellular RIPK1 suggested that almost 1 μM of the compound dissolved in blood plasma. In fact, the concentration of compound **22** at 1 h after oral administration showed a similar value, i.e. 2.031 μg ml^−1^ (4.4 μM) (Supplementary Table 3). In the same dosing samples, the compound in mouse blood reasonably inhibited monocyte cell deaths in a whole blood necroptosis assay (Fig. 6d).

These results suggest that the thermal stability of mouse RIPK1 in plasma is predictive marker to determine the target occupancy *in vivo*.

### *In vivo* mouse spleen and brain evaluation

The feasibility of this technology was evaluated with both spleen and brain extracted from mice orally dosed with compound. Both lipid and amino acid composition patterns are strikingly different for the brain and other tissues^34,35^. Therefore, extraction procedures of target protein have to be optimised for each tissue. As illustrated in Fig. 4b, dissected spleen and brain samples were divided into four approximately equal parts. The samples were independently treated at room temperature, or heated at 43, 45, and 47 °C for 8 min. The following sample procedures were similar for spleen and brain, but initial frozen and ultrasound homogenisation steps were required for brain sample preparation to improve RIPK1 protein extraction to the supernatant. In the same way as PBMC CETSA, thermal stability is estimated as supernatant per whole ratio.

**Figure 7:**
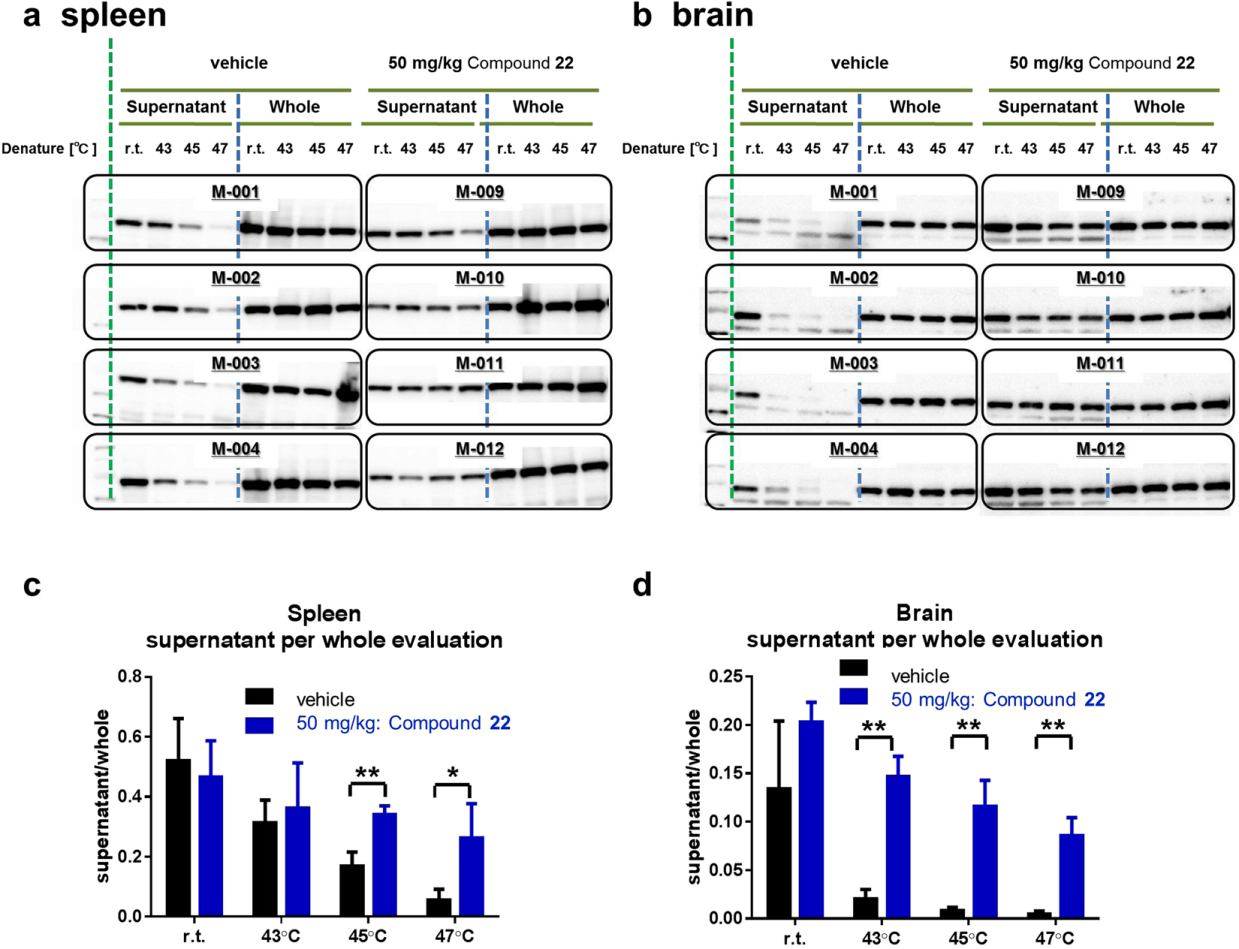
*In vivo* spleen and brain CETSA. T_agg_ experiments for both spleen and brain from orally administered compound **22**. Mice were divided into two groups each with four animals, vehicle control and experimental mice orally injected with 50 mg/kg compound **22**. One hour after drug administration, mice were euthanized, and spleen and brain were collected. Dissected spleen and brain samples were divided into four approximately equal parts. These were individually heated at room temperature, 43, 45, and 47 °C. Western blot analysis for isolated (**a**) spleen and (**b**) brain. All full-length Western blotting images are presented in Supplementary Fig. 11. (**c,d**) Based on the Western blot data (**a,b**), thermal stability is estimated from supernatant per whole ratio for (**c**) spleen and (**d**) brain. The chemiluminescence intensities of supernatant data are normalised to the response observed at the intensities of corresponding whole protein data. Data are provided as the average and standard error of the mean performed in four experimental animals. The results were significantly different from the vehicle group (Student’s *t*-test, *n = *4 per group, *Significant at *P* < 0.05, **Significant at *P* < 0.01). CETSA, cellular thermal shift assay; r.t., room temperature; T_agg_, aggregation temperature.

The spleen and brain samples were extracted from the same mice used in the previous *in vivo* mouse PBMC evaluation section, and were added to almost equal parts of pre-warmed PBS to avoid dilution of the reversible inhibitors. At 45 and 47 °C heat-denaturing conditions, 50 mg/kg compound **22**-treated spleen samples showed a significant increase in the thermal stability of mouse RIPK1 (Fig. 7a,c). Moreover, 50 mg/kg compound **22**-treated brain samples showed a significant increase in thermal stability of mouse RIPK1 at 43, 45, and 47 °C (Fig. 7b,d). The unbound concentrations in plasma and brain were 0.26 μM and 0.31 μM (Supplementary Tables 2 and 3), respectively. The unbound concentration in spleen was presumed to be almost equal to plasma, because unbound concentrations in plasma and brain are equal suggesting that unbound plasma concentration can be a surrogate of unbound concentration in tissues. These unbound concentrations were above the EC_50_ value of compound **22** in mouse L929 ITDRF assay (0.12 μM, Table 1). Therefore, our experimental procedure successfully evaluated the TE of mouse RIPK1 with *in vivo* spleen and brain samples.

## Discussion

Drug TE in a physiologically native condition is essential for preclinical and clinical drug development^4,5^. Although a number of reports have described that some potent irreversible inhibitors effectively exhibited cellular thermal shift in animal experiments^5^, there have been no reports, to our knowledge, suggesting that reversible drug leads show the promising CETSA effect in animal experiments accompanied by drug target occupancy. In this study, a 96-well-based semi-automated procedure and preparation methods for PBMCs, spleen, and brain were established, and the availability of SAR analysis with RIPK1 ITDRF assay was also demonstrated with cultured cells. Moreover, all our experimental procedures were performed under compound existence conditions before heating the samples, because the reversible compounds release from target proteins when the compound is diluted through the sample preparation processes. These experimental procedures demonstrate that CETSA is feasible to monitor the direct assessment of TE in animal studies using our recently developed reversible RIPK1 inhibitors.

To establish a robust and feasible CETSA for manipulating multiple samples, we developed a semi-automated system using automated pipetting and dispensing and a 96-well high-speed refrigerated centrifuge. The wash process for removing unwanted components in samples is a key element for reducing the background noise of detection systems, because both animal plasma and cell culture media have high protein concentrations. In fact, total plasma protein concentration is almost 100 mg ml^−1^; the capacity of Immun-Blot PVDF membrane retains 140–150 μg cm^−2^ based on BioRad supplier information^36^. This information suggests that almost 100-fold dilution is necessary to reduce the background. Using our semi-automated system, we are able to make almost 200-fold dilutions of protein concentrations and establish a feasible and reproducible method to evaluate SAR analysis in intricate physiological conditions with ITDRF experiments in a highly reproducible manner.

In order to understand the relationship among biophysical binding affinities under both *in vitro* recombinant and intracellular native conditions, and functional cellular activity, the 14 representative RIPK1 compounds were evaluated with three kinds of assay: human recombinant binding assay, HT-29 ITDRF assay, and HT-29 necroptosis assay. A significant positive linear correlation between ITDRF and the necroptosis assay was shown, with a similar positive correlation between ITDRF and the RIPK1 recombinant enzyme assay. The EC_50_ values of ITDRF were almost similar to those of the IC_50_ in the necroptosis assay and also showed similar IC_50_ values to the recombinant RIPK1 binding assay, except for GSK-compound **27**. GSK-compound **27** (furo[2,3-d]pyrimidine **27**) is distinct type 2 inhibitor occupying the ATP binding pocket with the kinase adopting a DLG-out conformation^28^. On the other hand, our developed compounds^22^ and Nec-1 are type 3 kinase inhibitors^37^ that bind to an allosteric pocket stabilizing a DLG-out conformation but not to the kinase hinge region and this results in excellent kinase selectivity. The reduction of binding affinity and cell-functional activity for GSK-compound **27** might be attributable to ATP competitiveness in the physiological environment of intracellular ATP concentration ranging from 0.5 to 5 mM^38^. These evaluations indicate that the RIPK1 ITDRF assay supports the interpretation of the physiological binding conditions within human cells.

In the case of mouse RIPK1, the EC_50_ values in mouse RIPK1-ITDRF assay of L929 cells for 7-oxo-2,4,5,7-tetrahydro-6H-pyrazolo[3,4-c]pyridine derivatives and Nec-1 also showed the almost similar Ki values on recombinant mouse RIPK1 except for GSK-compound **27**. This result is correlated with the assay results of human RIPK1. Furthermore, the order of inhibitory activity on recombinant enzyme assay is also correlated with two types of cellular functional assays, such as L-929 necroptosis assay and phospho (Ser-345) -MLKL ELISA assay. One question is that IC_50_ values in cellular assays were almost 10-fold lower than Ki values with mouse recombinant enzyme. In the case of L929 cells, TNF-α prominently causes necrosis instead of apoptosis, and this response is different from the other cell lines^39^. In fact, HT-29 cells require TNF-α, cIAP inhibitor, and a pan-caspase inhibitor to induce necroptosis^31^. Therefore, the necroptosis-inducible conditions are highly dependent on the kind of cell line. The L929 cells might be sensitive to necroptosis inducer and not require full target occupation of endogenous RIPK1.

To evaluate the feasibility of this methodology for *in vivo* animal experiments, we initially conducted the *ex vivo* analysis with PBMCs in addition to spike-injected tool compounds. There were no significant differences in the EC_50_ of ITDRFs for tool compounds among PBMCs derived from different mice. Since mouse ITDRF CETSA are applicable using *ex vivo* PBMCs, we applied this methodology to *in vivo* RIPK1 occupancy analysis for compound **22** orally dosing mice. Occupancy rate for each animal is calculated from supernatant per whole ratio by setting the full occupied control sample with spike-addition of high concentration of objective compounds. The 63.4% occupation of intracellular RIPK1 indicated that almost 1 μM compound dissolved in blood plasma and the concentration was similar values measured by the LC-MS method (4.4 μM). Therefore, the occupancy rate of *in vivo* mouse RIPK1 in PBMCs was successfully evaluated. To achieve the TE in animal tissues, the sample procedures have to be optimized for spleen and brain for improving RIPK1 protein extraction to the supernatant. Under optimized tissue disruption and thermal conditions, we successfully demonstrated the TE in both brain and spleen.

From a translational point of view, it is essential to show TE in humans. Without TE, it is difficult to conclude whether failures in clinical trials are due to poor drug potency or inappropriate selection of the target itself^1–3^. We have also confirmed the thermal stability of human RIPK1 when human PBMCs in plasma were treated at 1 μM of compound **22** for 30 min in the similar way to mouse PBMCs (Supplementary Fig. 7).

There are no consensus formulas to estimate the real binding affinities of the compound within cells based on ITDRF. Therefore, the establishment of formulas is a future challenge for this method.

This study demonstrates that CETSA is feasible to conduct comprehensive animal studies that enable to interpret the TE of a reversible kinase inhibitor in *in vivo* animal experiments, although a few groups have used this technology for tissue lysates^12^ and animal experiments treated with irreversible compounds^5^ and Michael acceptor inhibitor^10^. For clinical application, peripheral blood is easily accessible and widely used in research and toxicology. Our semi-automated system successfully evaluates drug TE and target occupation in both human and mouse PBMCs. Moreover, our optimised procedure for homogenisation of tissues allows us to monitor the TE in animal tissues, indicating the possibility of assessing TE in animal models. Our result indicates that CETSA will serve as an efficient tool for preclinical and clinical drug discovery.

## Methods

### Materials

Necrostatin-1 (Nec-1) was purchased from Sigma (M6006). The other chemical inhibitors including the fluorescent-labelled ligand 3-(3-((3-(4-amino-5-(4-(3-(2-fluoro-5-(trifluoromethyl)phenyl)ureido)phenyl)-7H-pyrrolo[2,3-d]pyrimidin-7-yl)propyl)amino)-3-oxopropyl)-5,5-difluoro-7,9-dimethyl-5H-dipyrrolo[1,2-c:2′,1′-f][1,3,2]diazaborinin-4-ium-5-uide were synthesised by Takeda Chemical Industries, Ltd^22^. Z-VAD-FMLK at a 20 mM stock solution in neat dimethyl sulfoxide (DMSO) was purchased from Promega (G7232) and Smac mimetic (AT-406) was from AdooQ BioScience (A11163-10). Recombinant mouse and rat TNF-α were purchased from Wako and R&D systems (410-MT-050 and 510-RT-050, respectively). The following antibodies were used for ELISA: mouse MLKL (MABC604, Millipore), mouse phospho-MLKL/S345 (ab196436, Abcam), and rabbit IgG HRP (711-035-152, Jackson ImmunoResearch). TMB was purchased from KPL (53-00-0302). The following antibodies were used for fluorescence-activated cell sorting (FACS) experiments: mouse anti-CD11b (553310, BD Biosciences; 1:200 dilution), and Ly-6G (127608, Biolegend; 1:400).

### Cell culture

Human colorectal adenocarcinoma (HT-29) and mouse L-cells NCTC 929 (L929) cells were acquired from ATCC (HTB-38 and CCL-1, respectively). HT-29 and L929 were cultured in McCoy’s 5 A medium (16600108, Invitrogen) and RPMI1640 medium (189-02025, WAKO), respectively, which were supplemented with 10% FBS, 100 U ml^−1^ penicillin, and 100 μg ml^−1^ streptomycin.

### *In vivo* mice experiment

C57BL/6 J (female, 10-week-old, Charles River Japan) were used for tissue CETSA experiments as well as *ex vivo* whole blood assay. The mice were housed on white chip. All procedures were performed in accordance with the standards for humane care, and treatment of research animal was approved by the Takeda Institutional Animal Ethics Committee (Approval No. 10805). RIPK1 inhibitor was suspended in 0.5% methyl cellulose and administered to mice orally. One hour after drug administration, mice were euthanized; blood was collected from the abdominal aorta with a heparin syringe (AY Pharma) and used for pharmacokinetic studies, FACS analysis, and CETSA assay; spleens and brains were extracted and used for pharmacokinetic studies and CETSA assay.

### Thermal shift experiments for culture cells and PBMCs

HT-29 and L929 cells were harvested and suspended with culture media containing 10% FBS to a cell density of 2 × 10^6^ cells ml^−1^. Mouse peripheral blood was collected from C57BL/6 J (female, 10-week-old, Charles River Japan) and PBMCs were isolated by density centrifugation over Lympholyte^®^-Mammal Cell Separation Media, Mammalian (CL5110, Cedarlane Laboratories) according to manual instructions. Briefly, approximately 500 μl of blood was carefully overlaid on 250 μl of the separation media so as not to disturb the layer. The samples were immediately spun at 800 *g* for 20 min at room temperature. After centrifugation, the buffy coat containing the PBMCs was collected with almost 350 μl of plasma to a cell density of approximately 1 × 10^6^ cells ml^−1^. Compounds were diluted from DMSO stock solutions at 10-fold concentrate in supplemental cell culture medium (final DMSO content 1%).

For the in-cell thermal aggregation curve experiments, the compound solution was mixed with 9-fold volume of the suspension of cells at approximately 2 × 10^6^ cells ml^−1^ and the cells were incubated for 30 min at 37 °C, 5% CO_2_. Cell suspension (culture cells, 100 μl/well; PBMCs, 30 μl/well) was then divided into each well of a 96-well PCR plate. The cells were transiently heated to different temperatures ranging from 40 to 60 °C for 3 or 8 min using a TaKaRa PCR Thermal Cycler Dice^®^ Gradient (TAKARA), followed by cooling at room temperature for 3 min. After the heating step, the plate was centrifuged at 800 *g* for 5 min at room temperature. In the case of culture cells, a volume of 70 μl of the supernatant was removed with MW508 (MS Technos). After that, 120 μl of PBS containing protease inhibitor cocktail (PBS wash buffer, 11873580001, Roche) was added to each well using MW508 for both culture cells and PBMCs. This wash procedure was repeated twice. After the final wash, 35 μl of PBS wash buffer was added to each well. The heat-treated cell suspensions were freeze-thawed three times using liquid nitrogen. For PBMC experiments, the PCR plate was gently vortexed and a volume of 12 μl of the cell lysate was transferred to a 96-well PCR plate as whole cell lysate samples. For both culture cells and PBMCs, the resulting cell lysates were centrifuged at 13,000 *g* for 30 min at 4 °C in order to separate the soluble proteins from the cell debris and aggregates in 96-well format using CR21G (HITACHI). A volume of 36 μl of supernatant containing the remaining soluble proteins was transferred to a 96-well PCR plate with MW508. Both whole and supernatant samples were analysed by sodium dodecyl sulfate polyacrylamide gel electrophoresis (SDS-PAGE) followed by Western blotting.

For the ITDRF in cell experiments, compounds were serially diluted to generate dose response curves. Cells were treated with each compound concentration and one vehicle as control in 100 μl of cell suspension in a 96-well PCR plate for 30 min at 37 °C, 5% CO_2_. The cell suspensions were heated at indicated temperatures. The heat-treated samples were treated as for the sample preparation and analysed with Western blotting following the procedure described above.

### Thermal shift experiments for *in vivo* tissue samples

Dissected spleen and brain samples were divided into four approximately equal parts in 2 ml Eppendorf tubes, and these tubes were put on ice. Within 15 min, the samples were added to 200 μl of pre-warmed PBS containing protease inhibitor. Immediately, the tubes were incubated for 8 min at regulated temperatures. The heat-treated brain samples were frozen using liquid nitrogen, and were homogenised with bead beaters (Shake-Master, Biomedical Science) using both 0.6 mm zirconium (ZS06-0001, Biomedical Science) and 3.0 mm stainless steel beads (SS30-0003, Biomedical Science) for 5 min under chilled conditions. After bead homogenisation, the samples were freeze-thawed three times, and then homogenised with ultrasonic tissue homogenisation (UH-50, SMT Company) on ice. As whole cell lysate samples, the sample tubes were vortexed and a volume of 100 μl of the homogenate was transferred to a 96-well PCR plate. The resulting homogenates were then centrifuged at 20,000 *g* for 20 min at 4 °C. The supernatant was removed from the tissue debris and aggregates. These samples were analysed with Western blotting. In the case of spleen samples, extraction efficiency of tissue RIPK1 was higher than that of the brain samples. Procedure for sample preparation of spleens excluded the processes of initial sample freezing and ultrasonic tissue homogenisation.

### *In vitro* necroptosis assay

The efficacies of RIP1 inhibitors were tested *in vitro* using HT-29 cells and L929 cells in a necroptosis assay^31^. For HT-29 necroptosis assay, frozen HT-29 cells were thawed and diluted to 1.5 × 10^5^ cells ml^−1^ in McCoy’s 5 A Medium (16600108, Invitrogen) supplemented with 10% FBS, 100 U ml^−1^ penicillin, and 100 μg ml^−1^ streptomycin. A 20 μl aliquot of cell suspension was added to each well of a 384-assay plate and the cell culture plates were incubated for 16–20 h at 37 °C, 5% CO_2_. After overnight incubation, 2.5 μl of inhibitor dissolved in culture medium was added to each well of the cell plates, and then each well was treated with 2.5 μl of necroptosis inducer containing rat TNF-α, AT-406, and zVAD-FMK solution at final concentrations of 200 ng ml^−1^, 10 μM, and 200 μM, respectively. Lidded plates were incubated for 16–20 h at 37 °C, 5% CO_2_. The next day, the supernatant of culture medium in each well was evaluated with CytoTox 96 Non-Radioactive Cytotoxicity Assay (G1780, Promega) according to the manufacturer’s instructions. The absorbance of the test plate was measured by 2103 EnVision Multilabel plate reader.

For mouse L929 necroptosis assay, L929 cells were seeded to a 96-assay plate at 3.0 × 10^4^ cells/120 μl in RPMI1640 medium (189–02025, WAKO) supplemented with 10% FBS, 100 U ml^−1^ penicillin, and 100 μg ml^−1^ streptomycin and the cells were cultured for 16–20 h at 37 °C, 5% CO_2_. After overnight incubation, the cells were treated with 15 μl of inhibitor containing media followed by the treatment with 15 μl of necroptosis inducer containing mouse TNFα and Z-VAD-FMK at final concentration of 20 ng ml^−1^ and 20 μM, respectively, and incubated for 4 h. The cytotoxicity assay was conducted in the same way as the HT-29 necroptosis assay. Percentage inhibition was calculated from the signal intensity of CytoTox assay by using the following formula; % inhibition = 100 − (A − X) × 100/(A − B).

A, no necroptosis inducer; B, necroptosis inducer; X, necroptosis inducer plus test inhibitor. IC_50_ values were calculated with XLfit software (IDBS) using a four-parameter logistic curve.

### SDS-Page, Western blot, and densitometry analysis

Lysates of culture cells, PBMCs, and tissue samples were mixed with 4-fold loading buffer (0.25 mM Tris-HCl, 8 w/v% SDS, 40 w/v% glycerol, 0.02 w/v% BPB, and 20 v/v% 3-mercapto-1,2-propandiol) and heated to denature the protein at 95 °C for 5 min. SDS-page was performed using standard protocol with BioRad Gel Croteropm TGX Any-KD 26 well (BioRad). Transfer of proteins to PVDF membranes was conducted with Tras Blot Turbo (Bio Rad) according to manufacturer’s protocols. Protein was detected using primary antibodies anti-monoclonal RIPK1 (610459, BD Transduction Laboratories™), anti-monoclonal RIPK2 (612348, BD Transduction Laboratories™), anti-RIPK3 polyclonal antibody (sc-135170, Santa Cruz Biotechnology) and β-actin antibody (#4967 L, Cell Signaling Technology); secondary antibodies anti-mouse IgG HRP conjugated antibody (#7076 S, Cell Signaling Technology) and anti-rabbit IgG HRP conjugated antibody (711-035-152, Jackson Immuno Research Laboratories). All membranes were blocked with blocking buffer and protein blots were imaged by ECL Pro (NEL121001EA, PerkinElmer) and detected with ImageQuant LAS 4000 (GE Healthcare). Protein bands in the membranes were quantified by ImageQuantTL v8.1.

### Data analysis

IC_50_ and EC_50_ values were calculated with either GraphPad Prism software (Version 5.03, Graph Pad Software) or XLfit software (IDBS) using a four-parameter logistic curve. All of the data are shown as mean ± SEM. For the T_agg_ shift and the ITDR experiments, data were analysed in GraphPad Prism using the Boltzmann sigmoid equation and the four-parameter logistic curve^4,24^. Student’s *t*-test was used to calculate *P* values with Microsoft Excel.

### Data availability

The authors declare that data supporting the findings of this study are available within the article.

## Electronic supplementary material

Supplementary information
