# Supplementary material for: CETSA quantitatively verifies in vivo target engagement of novel RIPK1 inhibitors in various biospecimens
Source: Sci Rep. 2017 Oct 12;7:13000. doi: 10.1038/s41598-017-12513-1 (PMC5638916; doi:10.1038/s41598-017-12513-1)
Supplement: Supplementary file 1 — Supplementary information [file 41598_2017_12513_MOESM1_ESM.pdf]

Supplementary Information

CETSA quantitatively verifies *in vivo* target engagement of novel RIPK1 inhibitors in various biospecimens

Tsuyoshi Ishii[1*], Takuro Okai[2], Misa Iwatani-Yoshihara[1], Manabu Mochizuki[1], Satoko Unno[2], Masako Kuno[2], Masato Yoshikawa[2], Sachio Shibata[3], Masanori Nakakariya[3], Takatoshi Yogo[2], & Tomohiro Kawamoto[1]

[1] Biomolecular Research Laboratories, Takeda Pharmaceutical Company Limited, 26-1, Muraoka-higashi 2-chome, Fujisawa, Kanagawa 251-8555, Japan. [2] Immunology Unit, Takeda Pharmaceutical Company Limited, 26-1, Muraoka-higashi 2-chome, Fujisawa, Kanagawa 251-8555, Japan. [3] Drug Metabolism & Pharmacokinetics Research Laboratories, Takeda Pharmaceutical Company Limited, 26-1, Muraoka-higashi 2-chome, Fujisawa, Kanagawa 251-8555, Japan.

Corresponding author: Tsuyoshi Ishii, Tel.: +81-3-3242-1256; Fax: +81-3-3278-2925

E-mail: tsuyoshi.ishii@takeda.com

Supplementary methods

Protein preparation. The human RIPK1 cDNA (NM_003804) coding 1-375 aa was cloned to have an N-terminal GST-His tag. GST-His-human RIPK1 (1-375) has two protease recognition sites and results in GST-(PreScission)-His-(TEV)-human RIPK1 (1-375). GST-His-human RIPK1 (1-375) baculovirus was generated using the BaculoDirect Baculovirus Expression System (Invitrogen) according to manufacturer's specifications. The baculovirus-infected Spodoptera frugiperda (Sf9) insect cells (2 L culture) were grown for 67 h at 27°C. A cell pellet was suspended in 200 ml lysis buffer (50 mM Tris-HCl, pH 8.0, 300 mM NaCl, 10 % glycerol, 1 mM DTT, and 5 U ml^{-1} Benzonase®). The suspension was then clarified by centrifugation at 14,000*g* for 30 min at 4°C. The supernatant was decanted from the insoluble pellet and bound to 3 ml of glutathione sepharose 4B (GE Healthcare) for 1.5 h at 4°C with gentle end-over-end rotation. The beads were then packed into a column and washed with lysis buffer (no Benzonase) and then eluted with 10 mM reduced glutathione in lysis buffer. Eluted fractions were concentrated to 8 ml and loaded onto a SDX200 26x600 SEC column (GE Healthcare) which had been equilibrated in 50 mM Tris-HCl, pH7.5, 150 mM NaCl, 10 % glycerol, and 1 mM DTT. Fractions identified by SDS-PAGE as containing protein of interest were pooled and concentrated to 7 ml. The protein concentration was determined by Bradford assay using BSA as a standard. The protein was stored at –80°C until use.

The mouse RIPK1 cDNA (NM_009068) coding 1-374 aa was cloned to have an N-terminal GST-His tag. GST-His-mouse RIPK1 (1-374) has two protease recognition sites

and results in GST-PreScission-His-TEV-mouse RIPK1 (1-374). The method for the production and purification of GST-His-mouse RIPK1 (1-374) protein was the same as that used for human RIPK1 (1-375) protein.

Competition recombinant enzyme binding assays. Time-resolved fluorescence resonance energy transfer (TR-FRET) assays were developed to evaluate interaction of novel test compounds at the ATP binding pocket of RIPK1, by competition with a fluorescent-labelled Type-2 RIPK1 inhibitor ligand. The fluorescent-labelled ligand 3-(3-((3-(4-amino-5-(4-(3-(2-fluoro-5-(trifluoromethyl)phenyl)ureido)phenyl)-7H-pyrrolo[2,3-d]pyrimidin-7-yl)propyl)amino)-3-oxopropyl)-5,5-difluoro-7,9-dimethyl-5H-dipyrrolo[1,2-c:2',1'-f][1,3,2]diazaborinin-4-ium-5-uide was used for human RIPK1 and mouse RIPK1 at final assay concentrations of 100 nM and 20 nM, respectively. Human GST-His-RIPK1 and Mouse GST-His-RIPK1 were used at final assay concentrations of 0.3 nM and 3 nM, respectively. Both the enzyme and ligand were prepared in solutions in 50 mM HEPES pH7.5, 10 mM NaCl, 50 mM $MgCl_2$, 0.5 mM DTT, and 0.02% CHAPS. To prepare the complex of GST-His-RIPK1 and LanthaScreen Tb-anti-GST (61GSTTLA, CisBio), GST-His-RIPK1 enzyme was pre-incubated for 1 h with a 533-fold diluted Tb-Anti-GST solution. 4 μl of inhibitor was dispensed to individual wells of a 384-well small volume white plate (784075, Greiner). Next, 4 μl of the fluorescent-labelled ligand was added to the test compounds. After that, 4 μl of mixture of GST-His-RIPK1 enzyme and Tb-anti-GST solution was added to each

reaction. The signal was measured 6 h later by EnVision (PerkinElmer). An excitation filter of 320 nm and emission filters of both 486 nm and 520 nm were used. Delay time and time windows were set to 100 μs and 200 μs, respectively. Inhibition rate was expressed as percent (%) inhibition of internal assay controls. Percentage inhibition was calculated from the signal intensity of FRET assay by using the following formula; % inhibition = (A – X) × 100 / (A – B)

A, DMSO control; B, 10 μM potent inhibitor (compound **18**, (3S)-3-(2-benzyl-3-chloro-7-oxo-2,4,5,7-tetrahydro-6H-pyrazolo[3,4-c]pyridin-6-yl)-5-methyl-2,3-dihydro-1,5-benzoxazepin-4(5H)-one); X. test inhibitor. For concentration response experiments, normalised data were fitted and IC_{50} values were calculated with XLfit software (IDBS) using a four-parameter logistic curve. The value of Ki (the inhibitory constant) was calculated based on Cheng-Prusoff equation: Ki = IC_{50}/(l + (ligand concentration) /(ligand-Kd))[1].

***In vitro* and *ex vivo* whole blood necroptosis assay.** For the isolation of mouse monocyte cells, mouse peripheral blood (~0.6 ml/mouse) was collected and PBMCs were isolated by density centrifugation over Ficoll-Paque Plus (GE Healthcare). Then, $CD11b^{+}$/$Ly\text{-}6G^{-}$ monocytes cells were isolated sequentially by using anti-Ly-6G MicroBead kit (130-092-332, Miltenyi) followed by anti-CD11b MicroBeads (130-049-60, Miltenyi), according to manufacturer’s recommendation.

For *in vitro* whole blood assay, 40 μl of peripheral blood were added to each well of the 96-well plate containing 12 μl of the appropriate concentration of inhibitors and 56 μl of RPMI1640 medium supplemented with 100 U ml^{-1} penicillin and 100 μg ml^{-1} streptomycin. After 30 min inhibitor incubation, 12 μl of mixture of necroptosis inducer including mouse TNF-α, AT-406, and zVAD-FMK was added to each well (final concentrations of 10 ng ml^{-1}, 1 μM, and 20 μM, respectively), and then the cells were incubated for 16–20 h at 37°C with 5% CO_2. After overnight incubation, cultured blood cells were mixed with Mouse BD Fc Block™ (553142, BD Biosciences) to block Fc receptor followed by the reaction with appropriate antibodies for 30 min on ice. Then, red blood cells were eliminated by adding 1x lysing buffer (555899, BD Biosciences) and the cells were washed once with PBS supplemented with 1% FBS. Cells were analysed on an SORP LSRII Fortessa or a SORP FACS Canto II flow cytometer (BD Biosciences).

For *ex vivo* experiments, 40 μl of peripheral blood was mixed with 68 μl of RPMI1640 and resulting 108 μl of diluted blood were added to each well of a 96-assay plate with 12 μl of mixture of necroptosis inducer mentioned above, and then the cells were incubated for 16–20 h at 37°C with 5% CO_2. The next day, the cells were collected and monocyte population was analysed by flow cytometry following the procedure described above. Percentage inhibition was calculated from the signal intensity of FACS analysis by using the following formula; % inhibition = 100 – (A – X) × 100 / (A – B); A, no necroptosis inducer; B, necroptosis inducer; X, necroptosis inducer plus test inhibitor. IC_{50} values were

estimated using a four-parameter logistic curve using GraphPad Prism 6.01 software (GraphPad Software Inc., La Jolla, CA, USA). All of the data are shown as mean ± SEM.

Mouse phospho-MLKL ELISA assay. Phospho-MLKL in L929 cells were detected by general sandwich ELISA. L929 cell lysates stimulated with necroptosis inducer were prepared in the same manner as for the *in vitro* necroptosis assay. The protein content of each cell lysates was adjusted to 200–300 μg ml^{-1} to be normalised. Then 100 μl of normalised samples were added onto 96-well ELISA plates (442404, Nunc) which had been coated with 100 μl of anti-mouse MLKL Ab (1 μg ml^{-1} in PBS) overnight at 4°C, and incubated for 1h at room temperature. Next, plates were incubated with 100 μl of anti-phospho-MLKL Ab (1 μg ml^{-1} in PBS supplemented with 1% BSA and 0.05% Tween20) for 1h at room temperature. Then, plates were reacted with 100 μl of anti-rabbit IgG HRP for 1h followed by detection with 100 μl of TMB. After stopping the reaction by adding 100 μl of 0.5 M H_2SO_4, plates were read on a spectrophotometer, Viento® (DS Pharma Biomedical) at A450.

Pharmacokinetic studies. Blood samples were centrifuged at 10,000*g* for 2 min, and the supernatant plasma was collected and stored at −80°C until analysis. Brain tissues were added to 10% (w/v) of PBS and homogenised using Tissue Lyser (QIAGEN). The concentrations of each compound in all the samples were determined using a UPLC system (Shimadzu, Japan) with a triple quadrupole mass spectrometry detection API-5000 (Applied Biosystems, U.S.A.)

equipped with a turbo ion spray interface.

Analyses for plasma protein binding (PPB) and brain tissue binding. These experiments used plasma and brain derived from male C57BL mice. The protein binding of each compound was determined by the equilibrium dialysis method with HTDialysis™ Teflon dialysis chambers and cellulose membranes (MWCO 6-8 kDa). Either plasma or brain homogenate was mixed with the compound solution at a final concentration of 1 μM. Dialysis was conducted against PBS in 8% CO_2 at 37°C for 16–20 h. The concentrations of compounds in both biological sample and PBS sides were determined by liquid chromatography/tandem mass spectrometry (LC-MS/MS, LC: Shimadzu UFLC, MS/MS: AB Sciex API4000). The unbound fraction in the biological sample was calculated as the ratio of the peak area of compounds from PBS side to those from either plasma or tissue side of the dialysis apparatus.

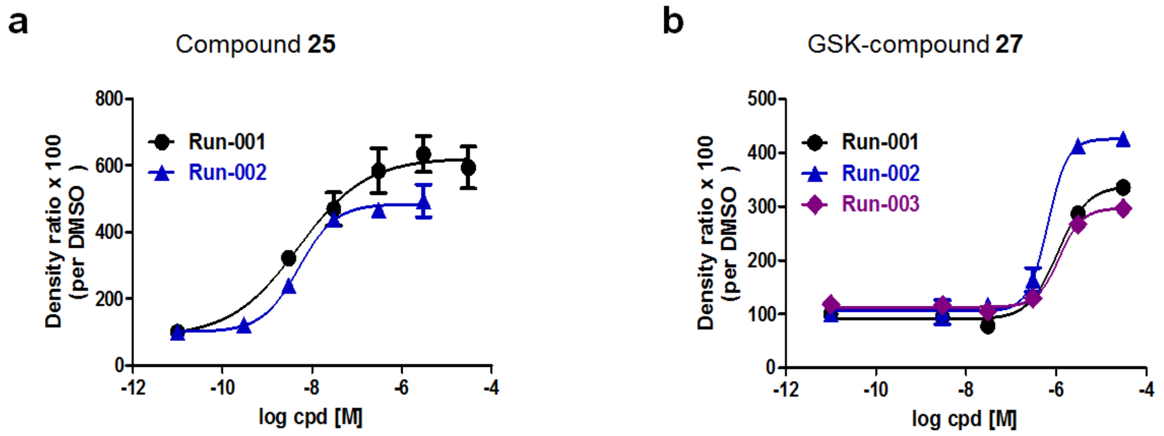

Supplementary Figure 1. Reproducibility analysis for ITDRF with HT-29 cells

(**a,b**) Reproducibility of ITDRF EC_{50} evaluation for compound **25** and GSK-compound **27** with HT-29 cells. Two (compound **25**) or three (GSK-compound **27**) independent results are shown in (**a**) and (**b**), respectively. The cell samples were heat treated at 47°C for 8 min. The experimental protocol is described in Methods. (**a**) The corresponding ITDRF for compound **25** run1 (black circle) and run 2 (blue triangle) resulted in EC_{50} of 4.9 nM (95% CI 1.0–24), 5.0 nM (95% CI 2.8–9.1), respectively. (**b**) The corresponding ITDRF for GSK-compound **27** run 1 (black circle), run 2 (blue triangle), and run 3 (purple diamond) result in EC_{50} of 1,100 nM (95% CI 700–1,700), 640 nM (95% CI 350–1,200), and 1,200 nM (95% CI 810–1,700), respectively. Data are provided as the average and standard error of the mean performed in duplicate.

CI, confidence interval; EC_{50}, half-maximal effective concentration; ITDRF, isothermal dose-response fingerprint.

a
HT-29 cells

DMSO	r.t.	42.4°C	49°C	56°C	59.9°C
total	2.2E+06	2.0E+06	1.9E+06	2.3E+06	2.1E+06
live	2.1E+06	2.0E+06	1.8E+06	2.2E+06	2.0E+06
dead	6.0E+04	4.0E+04	5.0E+04	4.0E+04	4.0E+04
viability	97%	98%	97%	98%	98%

30 µM Compound 22	r.t.	42.4°C	49°C	56°C	59.9°C
total	2.0E+06	2.0E+06	2.0E+06	2.1E+06	2.0E+06
live	2.0E+06	2.0E+06	1.9E+06	2.1E+06	2.0E+06
dead	6.0E+04	2.0E+04	4.0E+04	4.0E+04	2.0E+04
viability	97%	99%	98%	98%	99%

30 µM Compound 23	r.t.	42.4°C	49°C	56°C	59.9°C
total	2.0E+06	1.9E+06	2.0E+06	1.9E+06	1.9E+06
live	1.9E+06	1.9E+06	1.9E+06	1.8E+06	1.9E+06
dead	7.0E+04	7.0E+04	6.0E+04	4.0E+04	7.0E+04
viability	97%	97%	97%	98%	97%

30 µM Compound 25	r.t.	42.4°C	49°C	56°C	59.9°C
total	2.0E+06	1.9E+06	1.8E+06	2.0E+06	1.9E+06
live	1.9E+06	1.8E+06	1.8E+06	2.0E+06	1.8E+06
dead	5.0E+04	6.0E+04	4.0E+04	4.0E+04	6.0E+04
viability	97%	97%	98%	98%	97%

30 µM Nec-1	r.t.	42.4°C	49°C	56°C	59.9°C
total	2.1E+06	2.1E+06	1.9E+06	1.8E+06	2.1E+06
live	2.1E+06	2.0E+06	1.9E+06	1.8E+06	2.0E+06
dead	4.0E+04	4.0E+04	5.0E+04	4.0E+04	4.0E+04
viability	98%	98%	98%	98%	98%

30 µM GSK-compound 27	r.t.	42.4°C	49°C	56°C	59.9°C
total	1.9E+06	2.0E+06	1.9E+06	1.9E+06	2.0E+06
live	1.9E+06	1.9E+06	1.8E+06	1.8E+06	1.9E+06
dead	4.0E+04	5.0E+04	5.0E+04	6.0E+04	5.0E+04
viability	98%	97%	98%	97%	97%

b
L929 cells

DMSO	r.t.	42.4°C	49°C	56°C	59.9°C
total	1.6E+06	2.0E+06	1.7E+06	1.7E+06	1.6E+06
live	1.6E+06	1.9E+06	1.6E+06	1.6E+06	1.6E+06
dead	8.0E+04	9.0E+04	1.2E+05	7.0E+04	2.0E+04
viability	95%	95%	93%	96%	99%

30 µM Compound 22	r.t.	42.4°C	49°C	56°C	59.9°C
total	1.4E+06	1.8E+06	1.5E+06	1.6E+06	1.7E+06
live	1.4E+06	1.7E+06	1.4E+06	1.6E+06	1.5E+06
dead	5.0E+04	1.0E+05	1.0E+05	6.0E+04	1.6E+05
viability	97%	94%	94%	97%	91%

30 µM Compound 23	r.t.	42.4°C	49°C	56°C	59.9°C
total	1.8E+06	1.6E+06	1.6E+06	1.8E+06	1.5E+06
live	1.7E+06	1.5E+06	1.5E+06	1.7E+06	1.5E+06
dead	6.0E+04	7.0E+04	6.0E+04	7.0E+04	6.0E+04
viability	97%	96%	96%	96%	96%

30 µM Compound 25	r.t.	42.4°C	49°C	56°C	59.9°C
total	1.5E+06	1.9E+06	1.6E+06	1.7E+06	1.7E+06
live	1.5E+06	1.8E+06	1.5E+06	1.7E+06	1.6E+06
dead	7.0E+04	7.0E+04	5.0E+04	6.0E+04	6.0E+04
viability	96%	96%	97%	97%	97%

30 µM Nec-1	r.t.	42.4°C	49°C	56°C	59.9°C
total	1.6E+06	1.8E+06	1.7E+06	1.6E+06	1.8E+06
live	1.6E+06	1.8E+06	1.6E+06	1.5E+06	1.8E+06
dead	9.0E+04	5.0E+04	9.0E+04	8.0E+04	6.0E+04
viability	94%	97%	95%	95%	97%

30 µM GSK-compound 27	r.t.	42.4°C	49°C	56°C	59.9°C
total	1.5E+06	1.9E+06	1.7E+06	1.7E+06	1.6E+06
live	1.4E+06	1.8E+06	1.7E+06	1.6E+06	1.6E+06
dead	9.0E+04	1.1E+05	6.0E+04	9.0E+04	9.0E+04
viability	94%	94%	97%	95%	95%

Supplementary Figure 2. Trypan blue exclusion assay

The integrity of the cell membrane of both (**a**) HT-29 and (**b**) L929 cells during heating was performed with a trypan blue dye exclusion experiment. A 50 µl aliquot of 1.0×10^5 cells in culture medium were heated in the range of 40 to 60°C for 8 min in the presence of DMSO control or 10 µM compounds. After heating, samples were allowed to cool to room temperature for 3 minutes. A 50 µl aliquot of the heated cell suspension was mixed with a 50 µl aliquot of 0.4% trypan blue solution and subsequently analysed using a Countess Automated Cell Counter (Thermo Fisher Scientific). The data demonstrated that the cell

membrane integrities of both cell lines remain intact in the range of 40 to 60°C for 8 min because no statistically significant decrease in dye exclusion was observed compared to the initial dye exclusion capability of the cells.

DMSO, dimethyl sulfoxide.

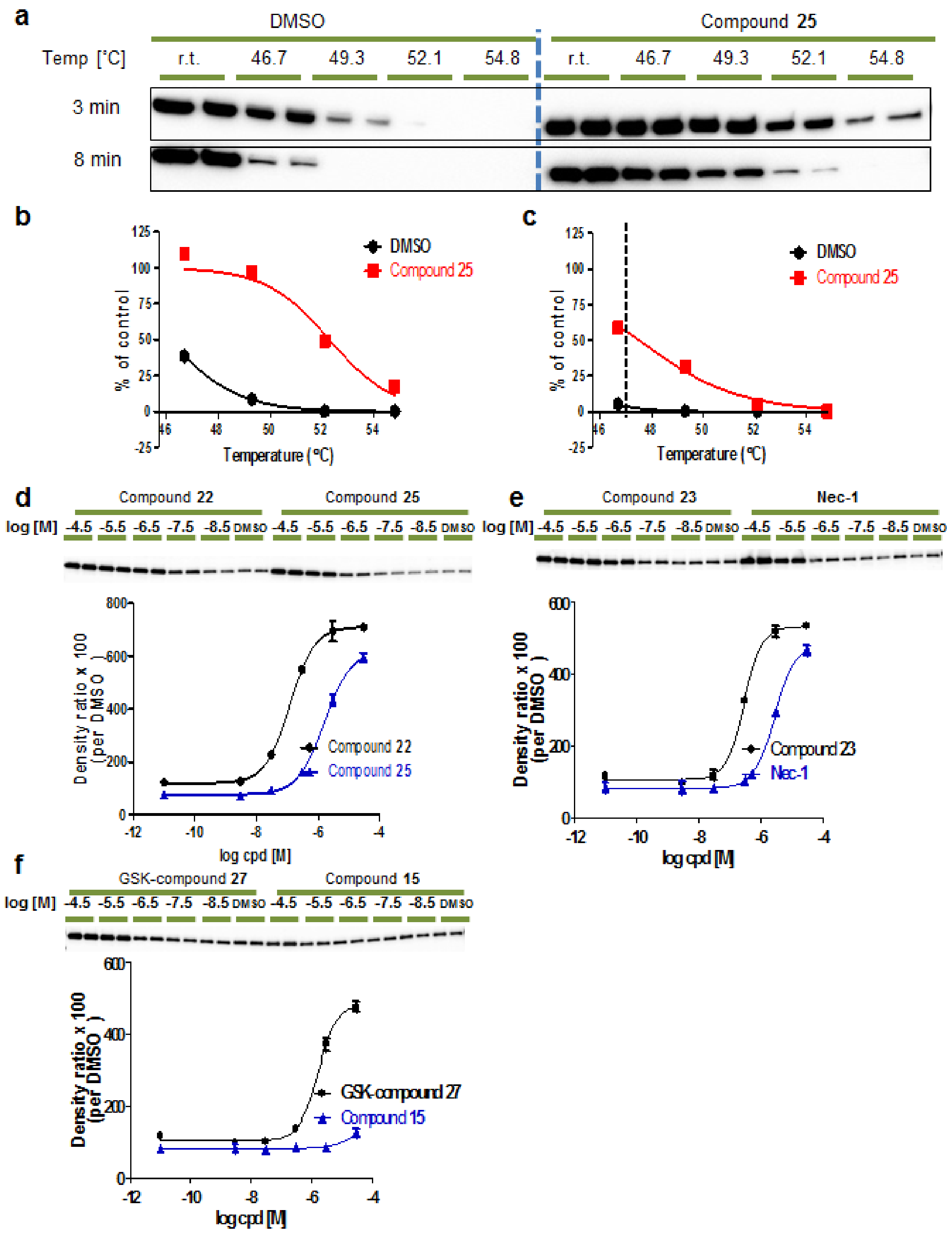

Supplementary Figure 3. Development of a CETSA for mouse RIPK1 with L929 cells

(**a**) Western blotting analysis of T_{agg} for mouse RIPK1 in L929 cells. The cells were heated in the range of room temperature to 54.8°C for either 3 min or 8 min in the presence of 10% FBS and allowed to cool to room temperature for 3 minutes. (**b,c**) T_{agg} curves for RIPK1 in

L929 cells in the presence of DMSO (0.1%) (black circle) and 10 μM compound **25** (red square). Evaluation of (**b**) 3 min and (**c**) 8 min denature conditions was performed to verify the optimum conditions. All data were normalised to the response observed at DMSO-treated condition at room temperature. The T_{agg} shifts were analysed using the Boltzmann sigmoid equation. The vertical dotted line is at 47°C for 8 min denature, the experimental condition selected for the ITDRF assay. Data are provided as the average and SEM performed in duplicate. (**d–f**) ITDRF of representative RIPK1 inhibitors at 47°C for 8 min denature based on raw data from the Western blotting chemiluminescence readings. The chemiluminescence data are shown above the graphs. ITDRF lines are fitted with a four-parameter logistic curve. The corresponding EC_{50} values of ITDRF resulted in: compound **22** (**d**, black circle) = 120 nM (95% CI 91–160); compound **25** (**d**, blue triangle) = 1600 nM (95% CI 1,200– 2,100); compound **23** (**e**, black circle) = 290 nM (95% CI 250–340); Nec-1 (**e**, blue triangle) = 2,800 nM (95% CI 2,200– 3,600); GSK-compound **27** (**f**, black circle) = 2,600 nM (95% CI 1,200–2,200); compound **15** (**f**, blue triangle) >30,000 nM. Data are provided as the average and SEM performed in duplicate.

CETSA, cellular thermal shift assay; CI, confidence interval; DMSO, dimethyl sulfoxide; EC_{50}, half-maximal effective concentration; ITDRF, isothermal dose-response fingerprint; RIPK1, receptor interacting protein 1 kinase; SEM, standard error of the mean.

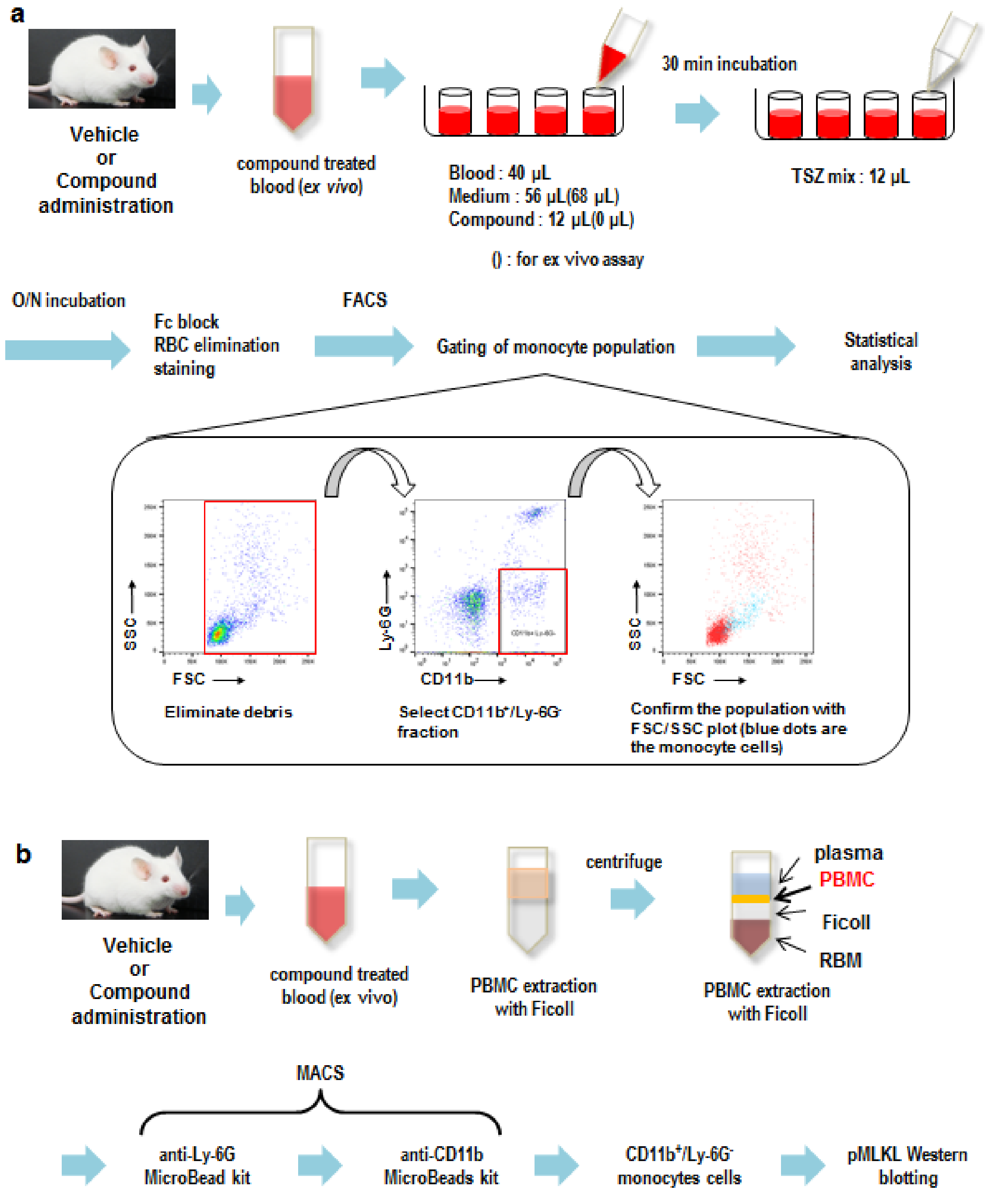

Supplementary Figure 4. Experimental procedure for both *in vitro* and *ex vivo* whole blood necroptosis assay

(**a**) Illustration of whole blood necroptosis assay flow in *in vitro* and *ex vivo*. Briefly, peripheral blood was collected from normal mice (*in vitro* assay) or mice treated with vehicle or compounds (*ex vivo* assay) and added to each well of the 96-well plate as indicated

(parenthetical numbers are for *ex vivo* assay). With (*in vitro*) or without (*ex vivo*) 30-min incubation with each compound, the mixture of necroptosis inducer including (TSZ mix) was added to each well and then the cells were incubated for 16–20 h at 37°C. Next day, cultured blood cells were stained with anti-CD11b and anti-Ly-6G antibodies after Fc block and elimination of red blood cells, and applied to an FACS Canto II flow cytometer. Data were analysed with FlowJo software and the gating of monocyte population was indicated. (**b**) Isolation of CD11b+/Ly6G- monocyte cells is described. Mouse peripheral blood was collected and PBMCs were isolated by density centrifugation over Ficoll-Paque Plus. Then, CD11b+/Ly-6G- monocytes cells were isolated sequentially by using anti-Ly-6G MicroBead kit followed by anti-CD11b MicroBeads, according to manufacturer's recommendations. Details are given in Methods.

PBMC, peripheral blood mononuclear cell.

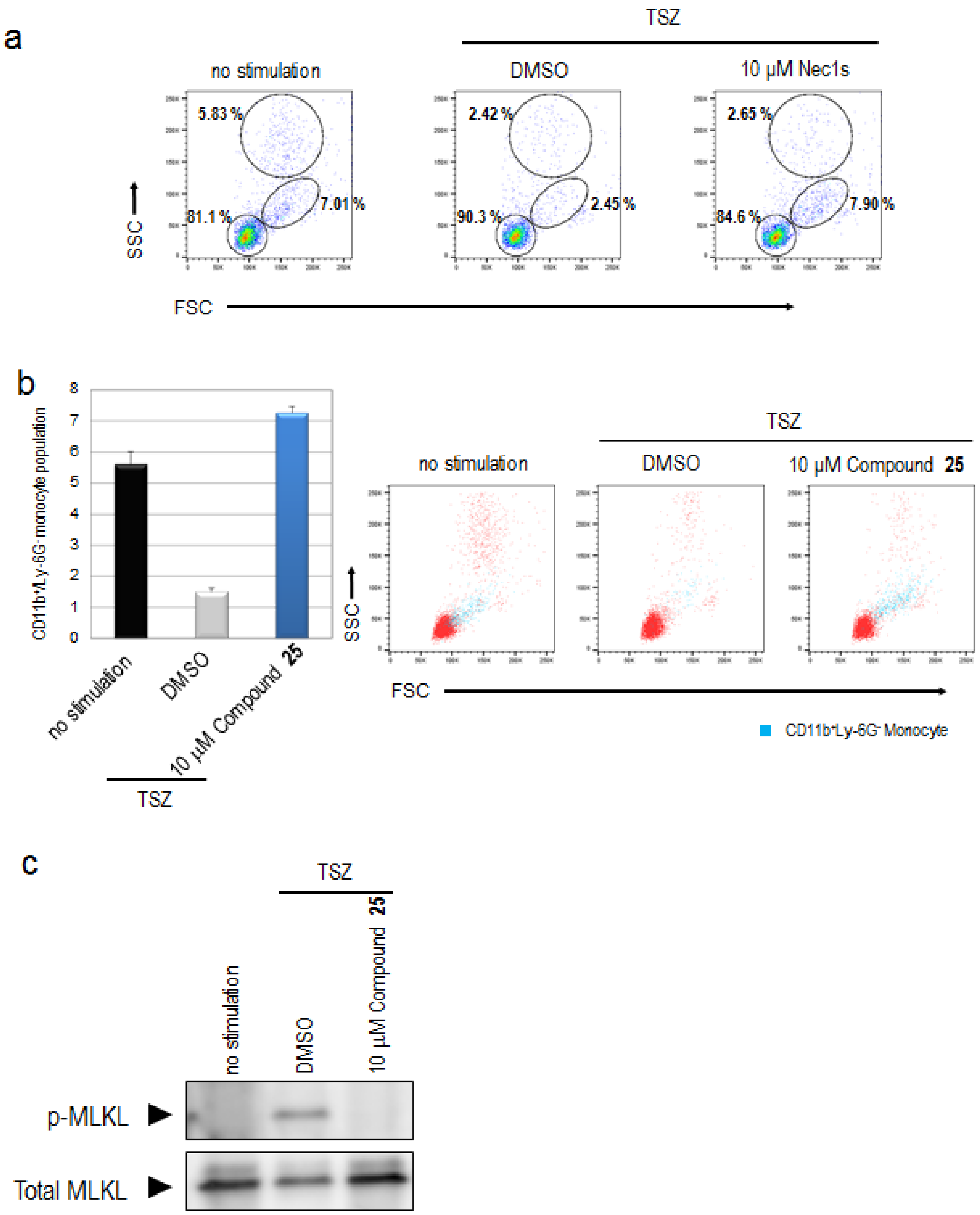

Supplementary Figure 5. Establishment of *in vitro* whole blood necroptosis assay

(**a**) To examine whether some cell populations are sensitive to necroptosis inducer, leucocytes, monocytes, and neutrophil fractions in cultured whole blood were assessed when TSZ was added. Since leucocytes, monocytes, and neutrophil fraction can roughly be gated from FSC and SSC scattered plot, each fraction was gated and % frequency was calculated in each

treatment (no stimulation, TSZ stimulation with DMSO control, and TSZ stimulation with 10 mM Nec-1). As a result, the monocyte and neutrophil fraction was decreased, in which fractions only the monocyte fraction was restored by the treatment of RIPK1 inhibitor. (**b**) Monocyte cell number was counted as CD11b+/Ly-6G- population by FACS staining for further confirmation, since CD11b+ fraction contains Ly-6G+ neutrophil population. The left-hand graph indicates the CD11b+Ly-6G- monocyte population which was calculated from flow cytometry analysis. Representative plots are indicated in the right-hand panels in which CD11b+/Ly-6G- population are shown as blue dots. Significant reduction in monocyte number was observed with TSZ stimulation, which was completely rescued with compound **25** treatment. (**c**) CD11b+Ly-6G- monocyte cells were isolated as described in Supplementary Fig. 4b and analysed by immunoblotting using the indicated antibodies. Each lane contains 1–2 μg proteins. MLKL phosphorylation (p-MLKL), a necroptosis marker, was increased with TSZ treatment, which was completely inhibited by compound **25** treatment, indicating that the monocyte number loss in (**b**) was due to necroptotic cell death. Based on these data, we concluded that monocyte population can be used to assess potency of RIPK1 inhibitor in whole blood necroptosis assay.

DMSO, dimethyl sulfoxide; RIPK1, receptor interacting protein 1 kinase; TSZ, necroptosis stimulator.

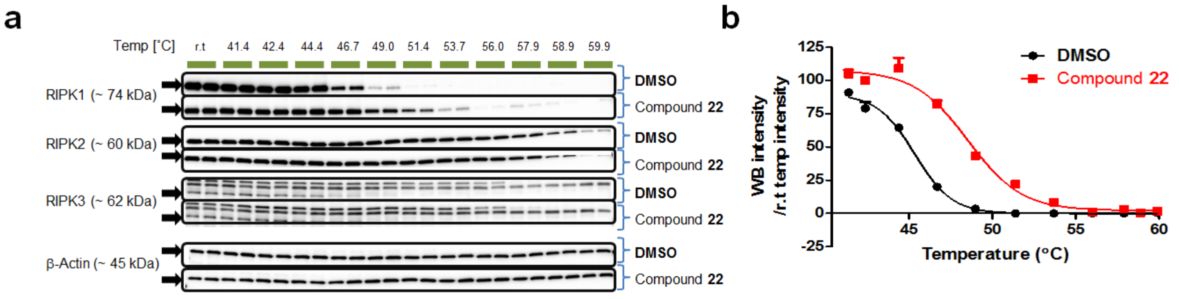

Supplementary Figure 6. Characterisation of compound 22 using CETSA

(**a**) CETSA was performed on HT-29 cells in the presence of DMSO (0.1%) or 10 µM compound **22**. The cell samples were heat treated at 47°C for 8 min. The stabilising effects of compound **22** on RIPK1, RIPK2, RIPK3, and β-actin at different temperatures were evaluated with Western blot analysis. (**b**) Based on the chemiluminescence result of RIPK1 shown in (**a**), T_{agg} curves for RIPK1 in HT-29 cells in the presence of DMSO (0.1%) (black closed circle) and compound **22** (red square). All data were normalised to the response observed at DMSO-treated condition at room temperature. The T_{agg} shift was analysed using the Boltzmann sigmoid equation. Data are provided as the average and SEM performed in duplicate.

CETSA, cellular thermal shift assay; DMSO, dimethyl sulfoxide; EC_{50}, half-maximal effective concentration; ITDRF, isothermal dose-response fingerprint; RIPK1, receptor interacting protein 1 kinase; SEM, standard error of the mean.

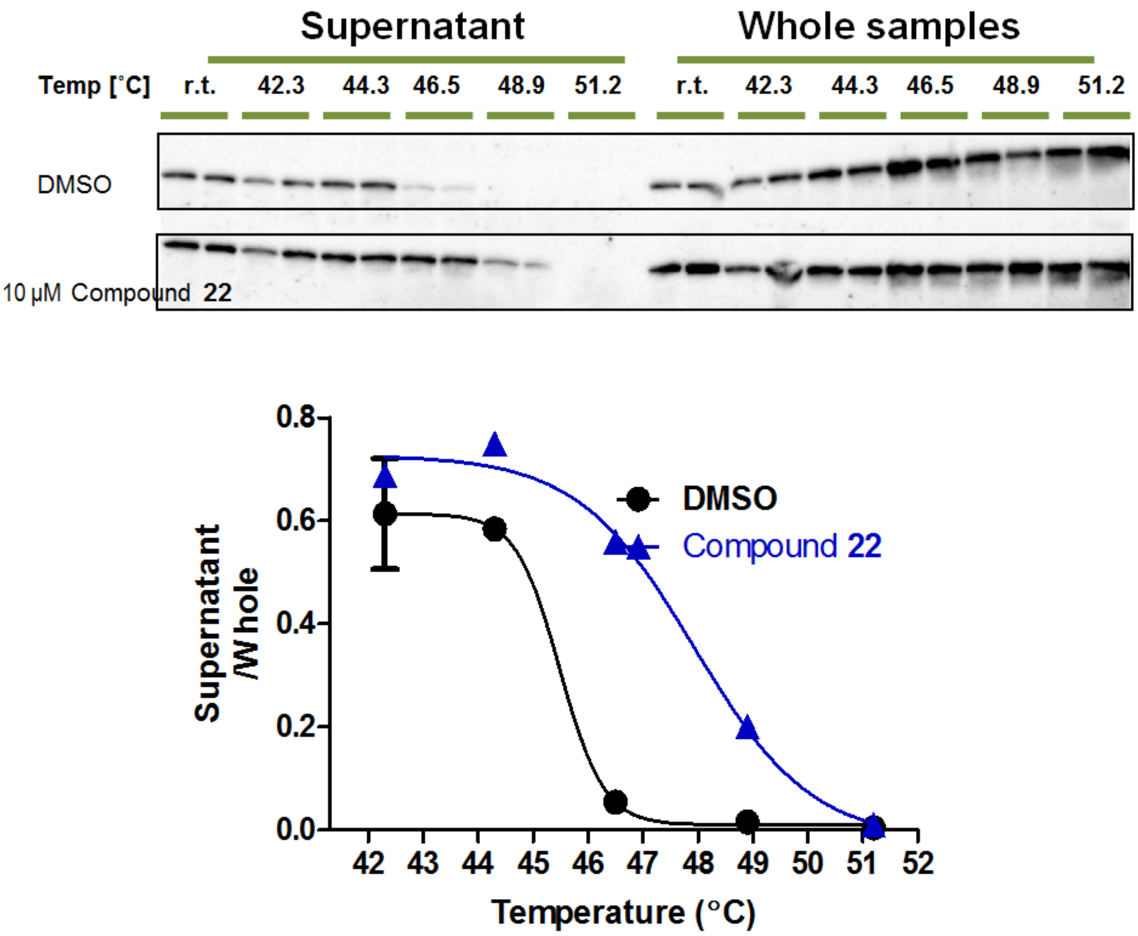

Supplementary Figure 7. T_{agg} evaluation for human RIPK1 in PBMCs suspended in human blood

Western blotting analysis of T_{agg} for RIPK1 in PBMCs suspended in human blood. A suspension of human PBMCs was heated in the range of room temperature to 51.2°C for 8 min in the presence of either DMSO (0.1%) or 1 μM compound 22, and allowed to cool to room temperature for 3 minutes. T_{agg} curves for RIPK1 in the presence of DMSO (0.1%) (black circle) and 1 μM compound **22** (blue triangle). All data were normalised to the response observed at DMSO-treated condition at room temperature. The T_{agg} shifts were analysed using the Boltzmann sigmoid equation. The chemiluminescence intensities of supernatant data were normalised to the response observed at the corresponding whole protein.

Data are provided as the average and SEM performed in duplicate.

DMSO, dimethyl sulfoxide; PBMC, peripheral blood mononuclear cells; RIPK1, receptor interacting protein 1 kinase; SEM, standard error of the mean; T_{agg}, aggregation temperature.

a DMSO (Fig. 2b)

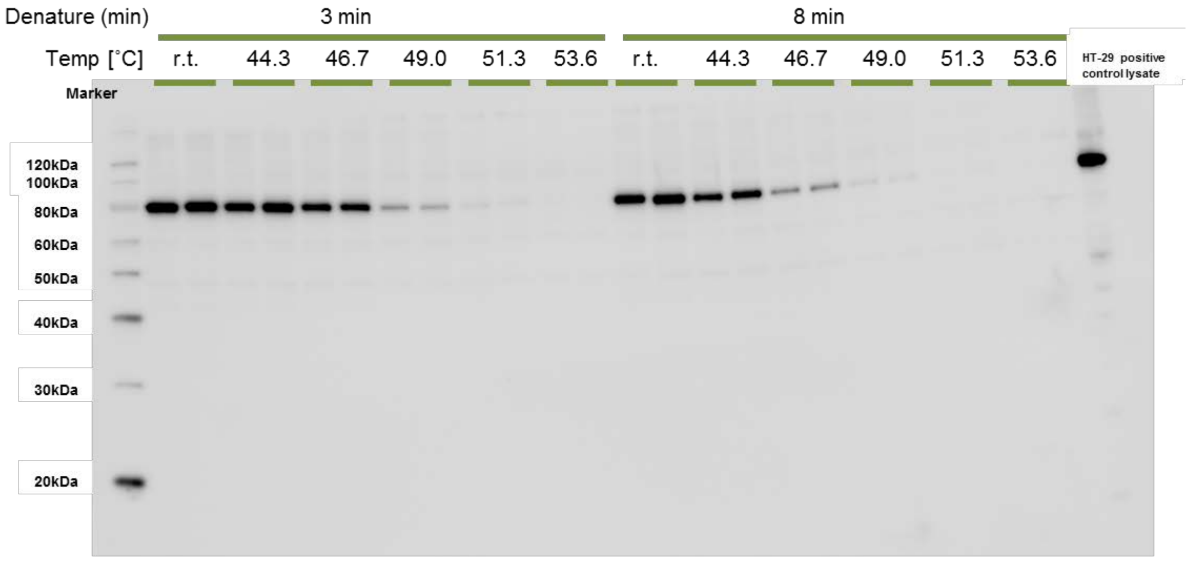

b Compound **15 (Fig. 2b)**

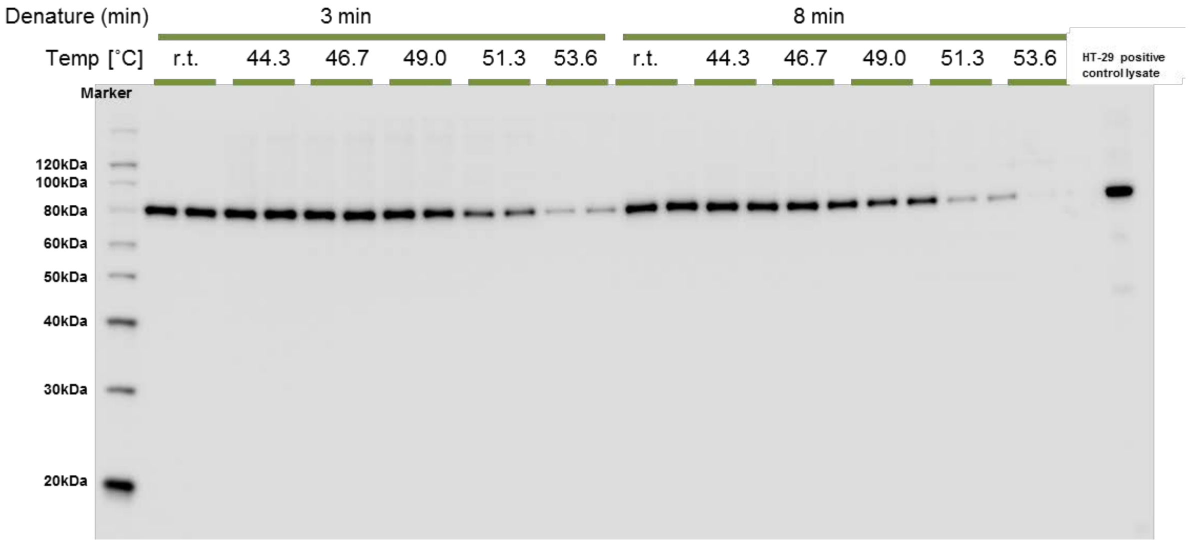

Supplementary Figure 8. All full-length Western blotting images in Figure 2

c Compound **25 (Fig. 2b)**

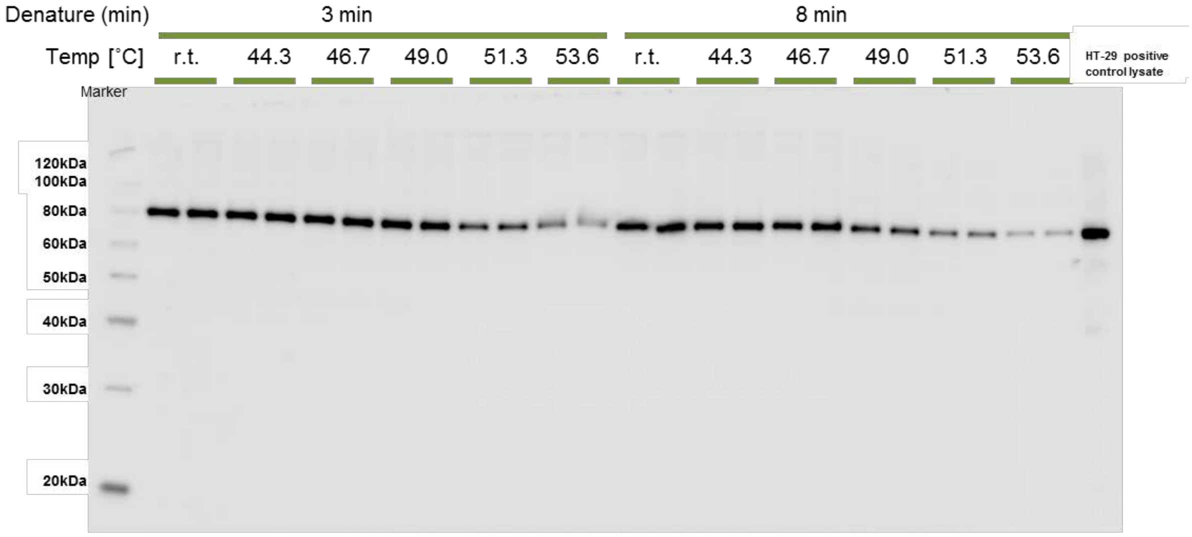

d GSK-compound **27 (Fig. 2b)**

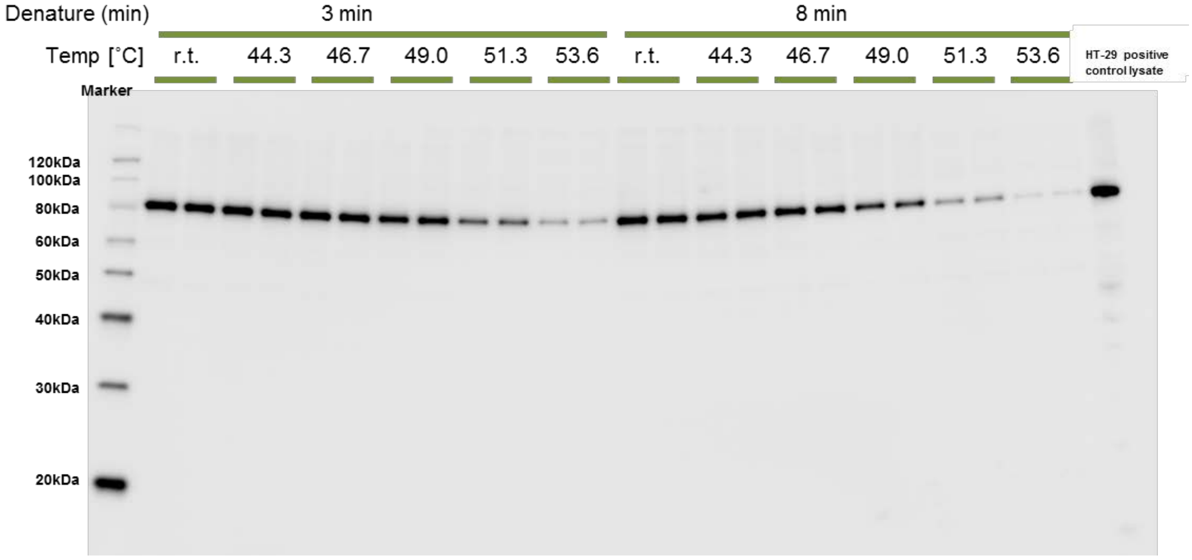

Supplementary Figure 8. All full-length Western blotting images in Figure 2 (continued)

e Compound **25** (Fig. 2c)

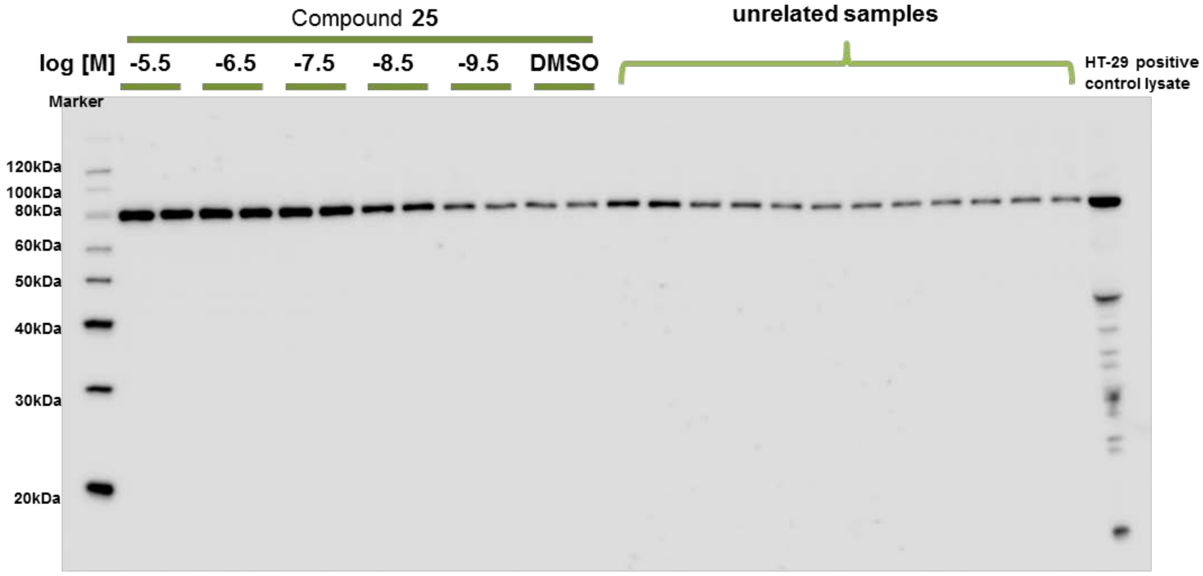

f Nec-1 (Fig. 2c)

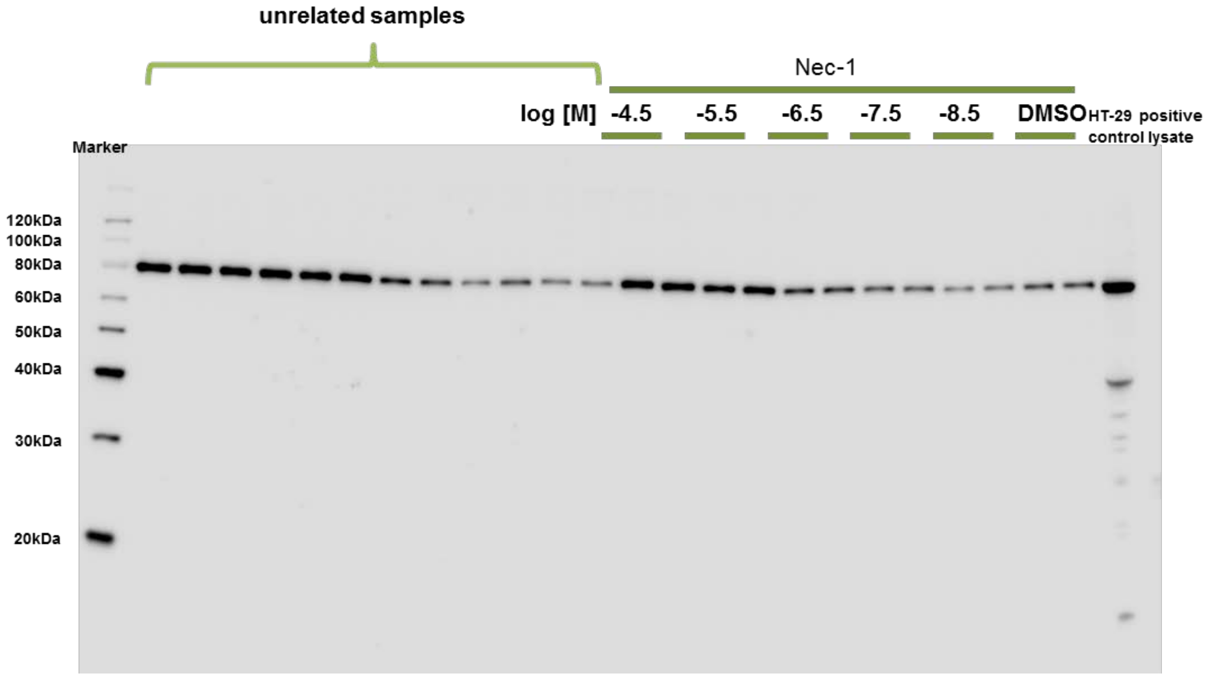

Supplementary Figure 8. All full-length Western blotting images in Figure 2 (continued)

g Compound **22**, GSK-compound**27** **(Fig. 2d)**

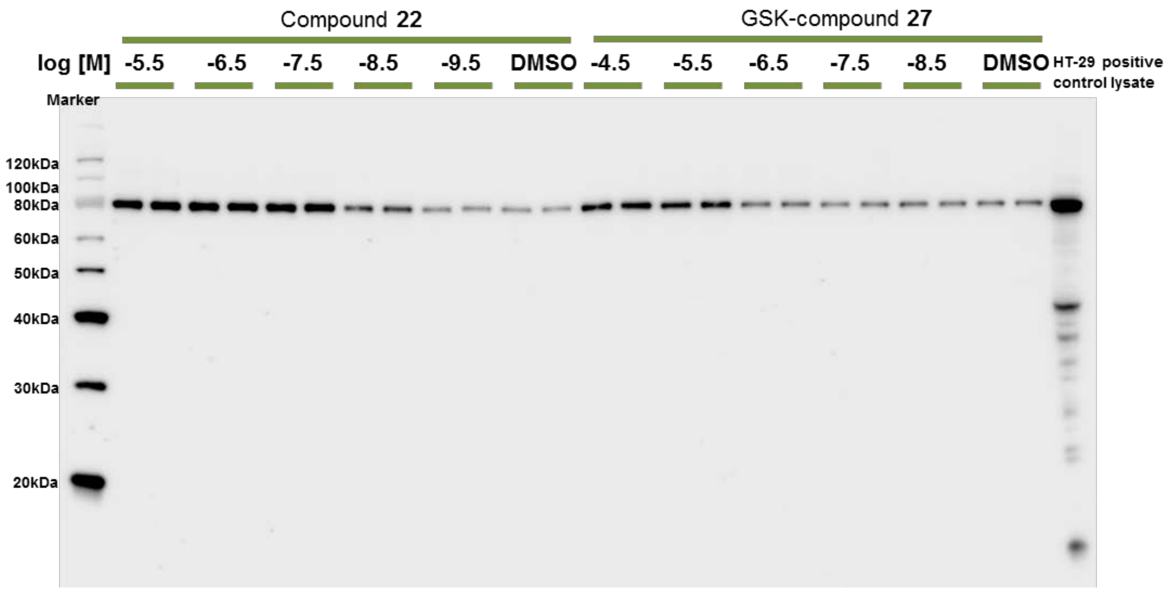

Supplementary Figure 8. All full-length Western blotting images in Figure 2 (continued)

Full images of figure 2b are shown in (a) DMSO, (b) compound **15**, (c) compound **25**, and (d) GSK-compound **27**. Full images of figure 2c are shown in (e) compound **25** and (f) Nec-1. Full image of figure 2d is shown in (g) compound **22** & GSK-compound **27**. Total cell extracts from HT-29 cells serve as a positive control.

a Compound **22, mouse-1**

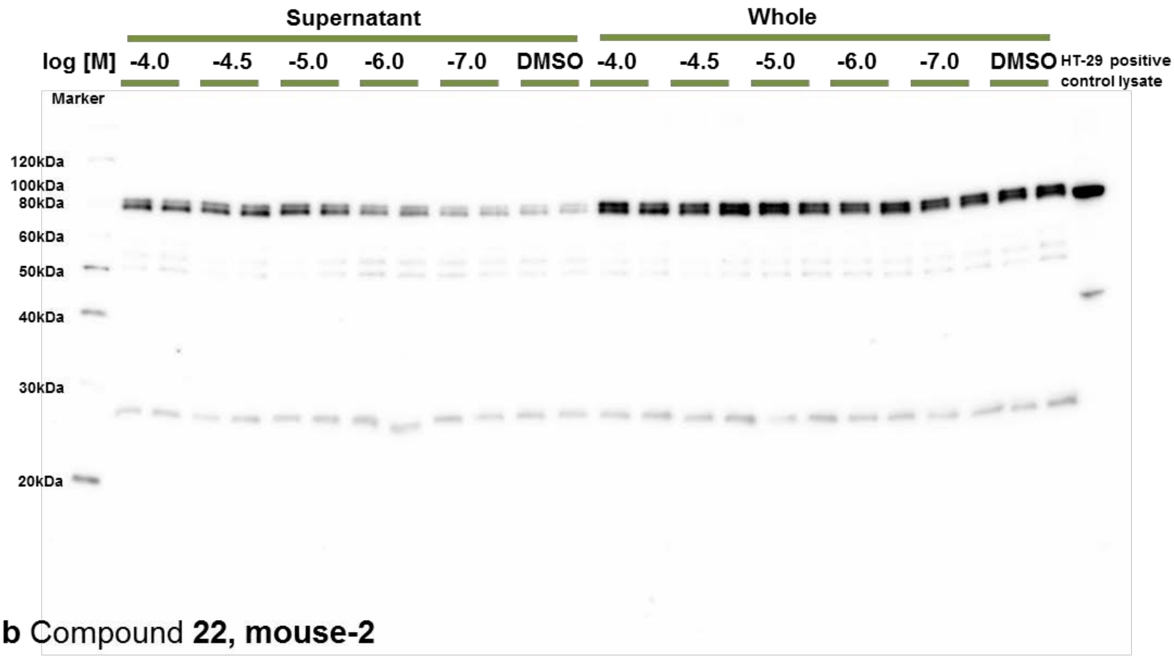

b Compound **22, mouse-2**

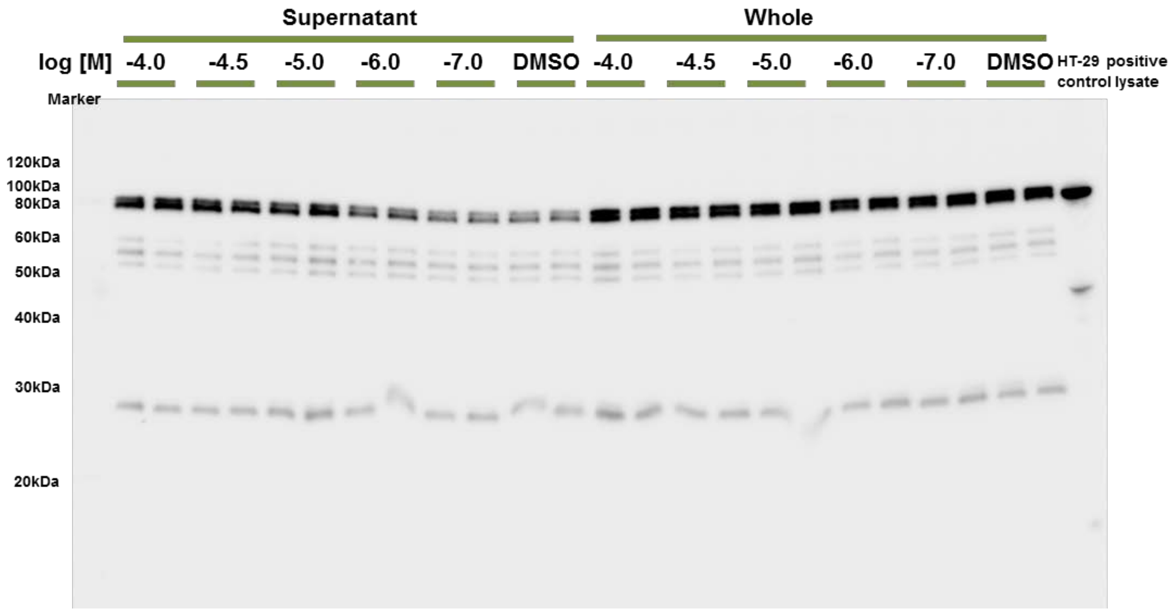

Supplementary Figure 9. All full-length Western blotting images in Figure 5

c Compound **22, mouse-3**

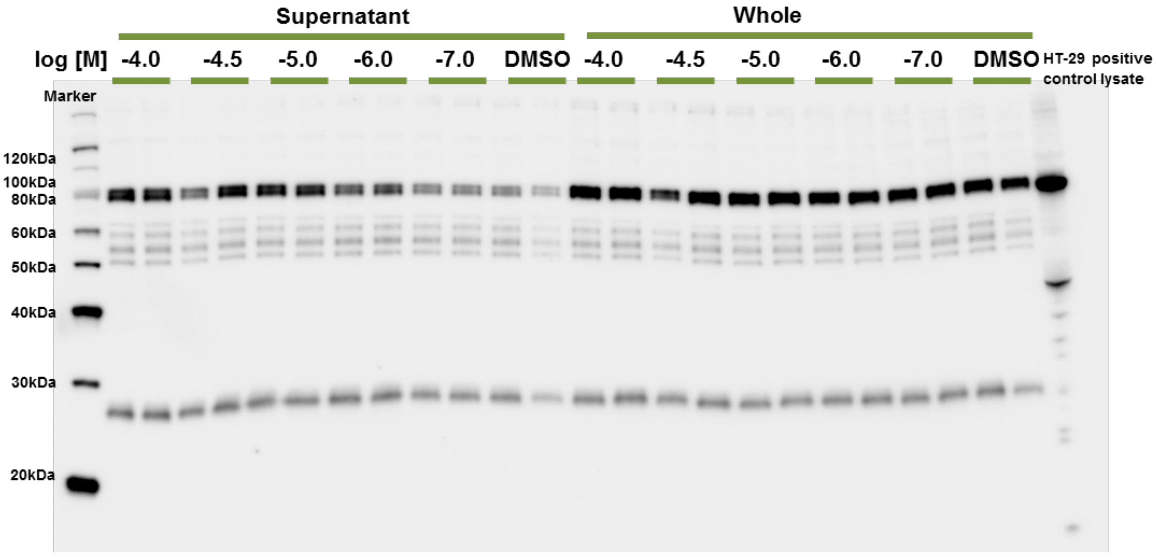

Supplementary Figure 9. All full-length Western blotting images in Figure 5 (continued)

d Compound **25, mouse-1**

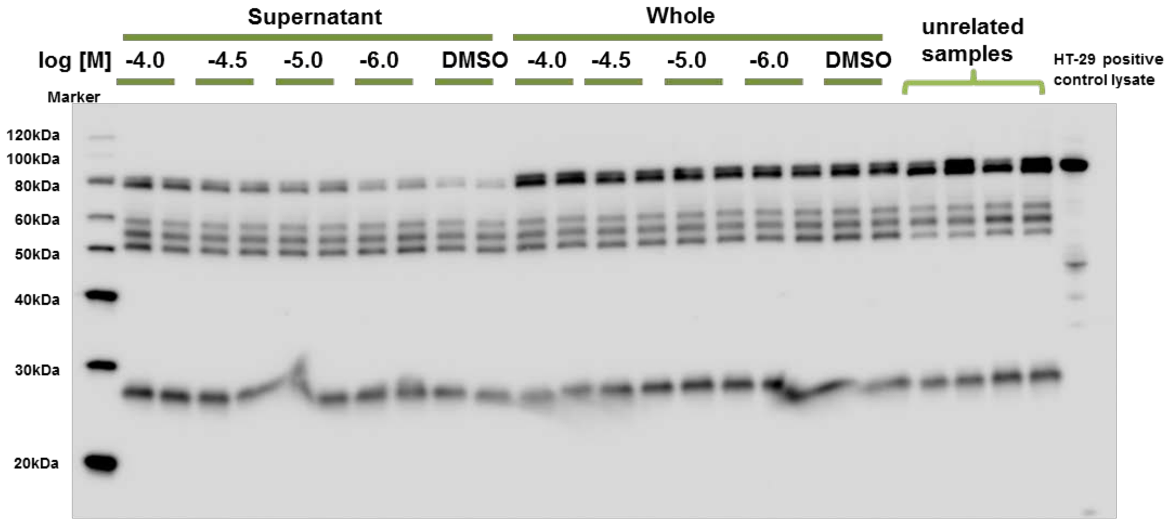

e Compound **25, mouse-2**

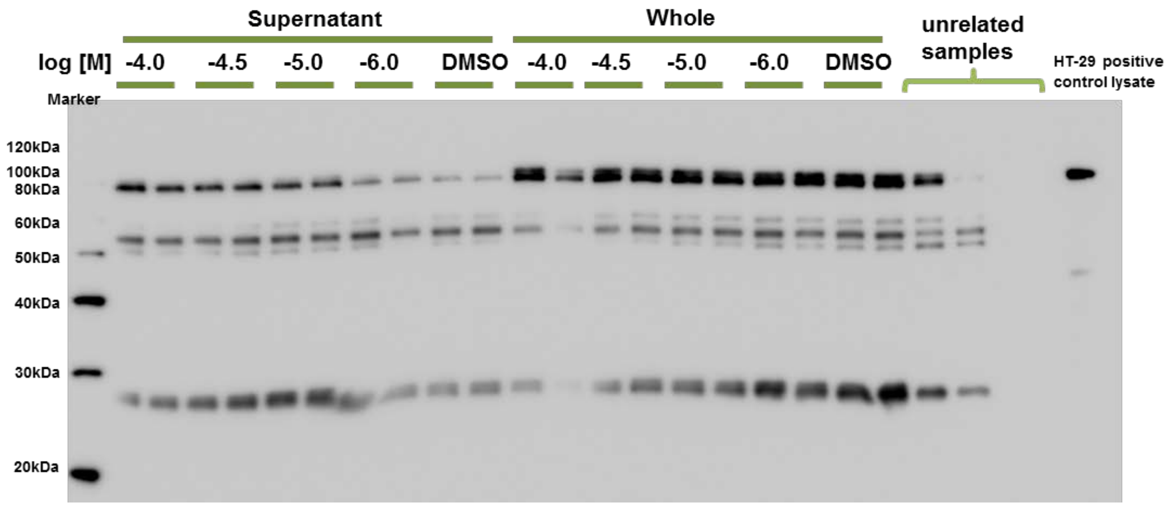

Supplementary Figure 9. All full-length Western blotting images in Figure 5 (continued)

Full images of figure 5a are shown in (a) compound **22** treated mouse-1, (b) compound **22** treated mouse-2, and (c) compound **22** treated mouse-3. Full images of figure 5b are shown in (d) compound **25** treated mouse-1, and (e) compound **25** treated mouse-2. Total cell extracts from HT-29 cells serve as a positive control.

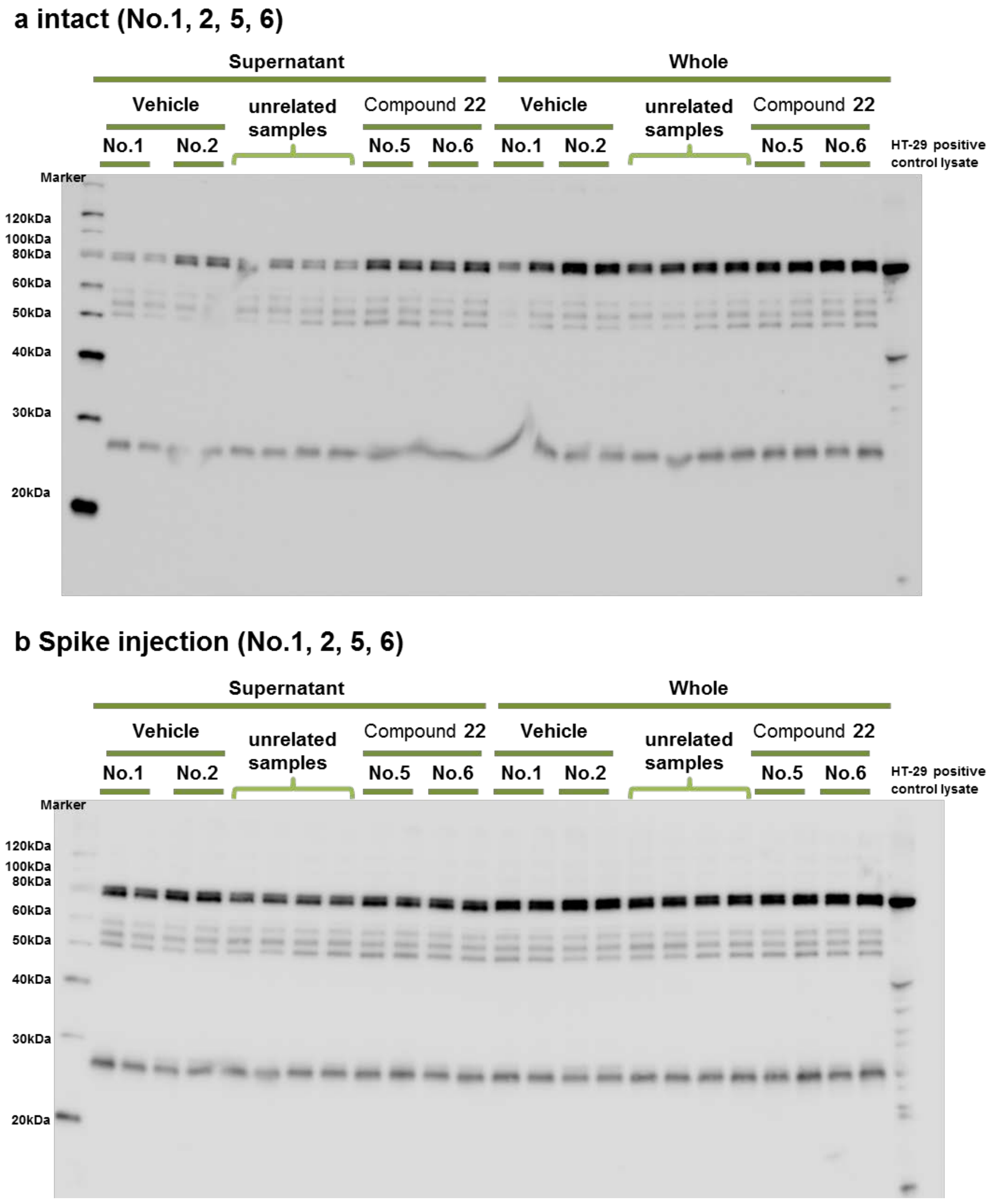

Supplementary Figure 10. All full-length Western blotting images in Figure 6

c intact (No.3, 4, 7, 8)

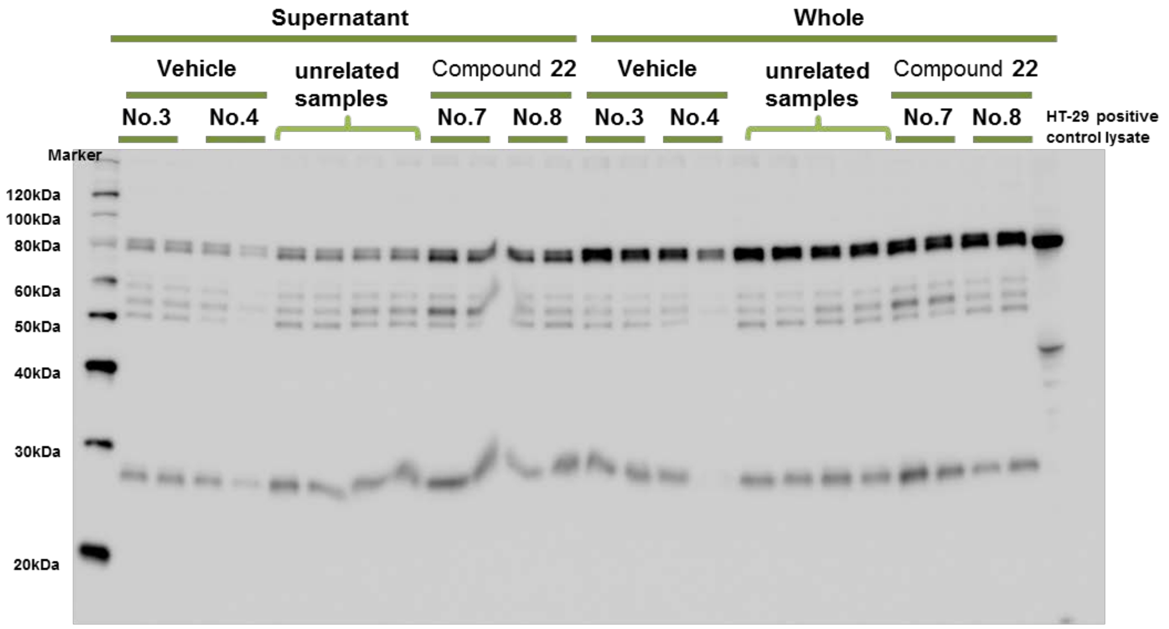

d Spike injection (No.3, 4, 7, 8)

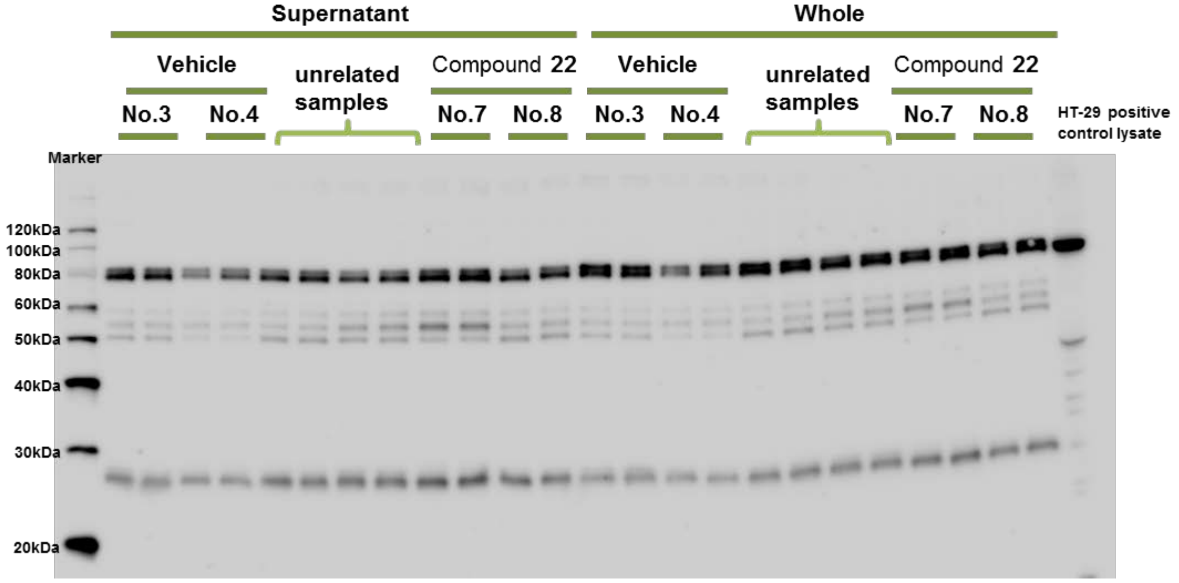

Supplementary Figure 10. All full-length Western blotting images in Figure 6 (continued)

Full images of figure 6a are shown in (a) intact (No.1, 2, 5, 6), (b) Spike injection (No.1, 2, 5, 6), (c) intact (No.3, 4, 7, 8), and (d) Spike injection (No.3, 4, 7, 8). Total cell extracts from HT-29 cells serve as a positive control.

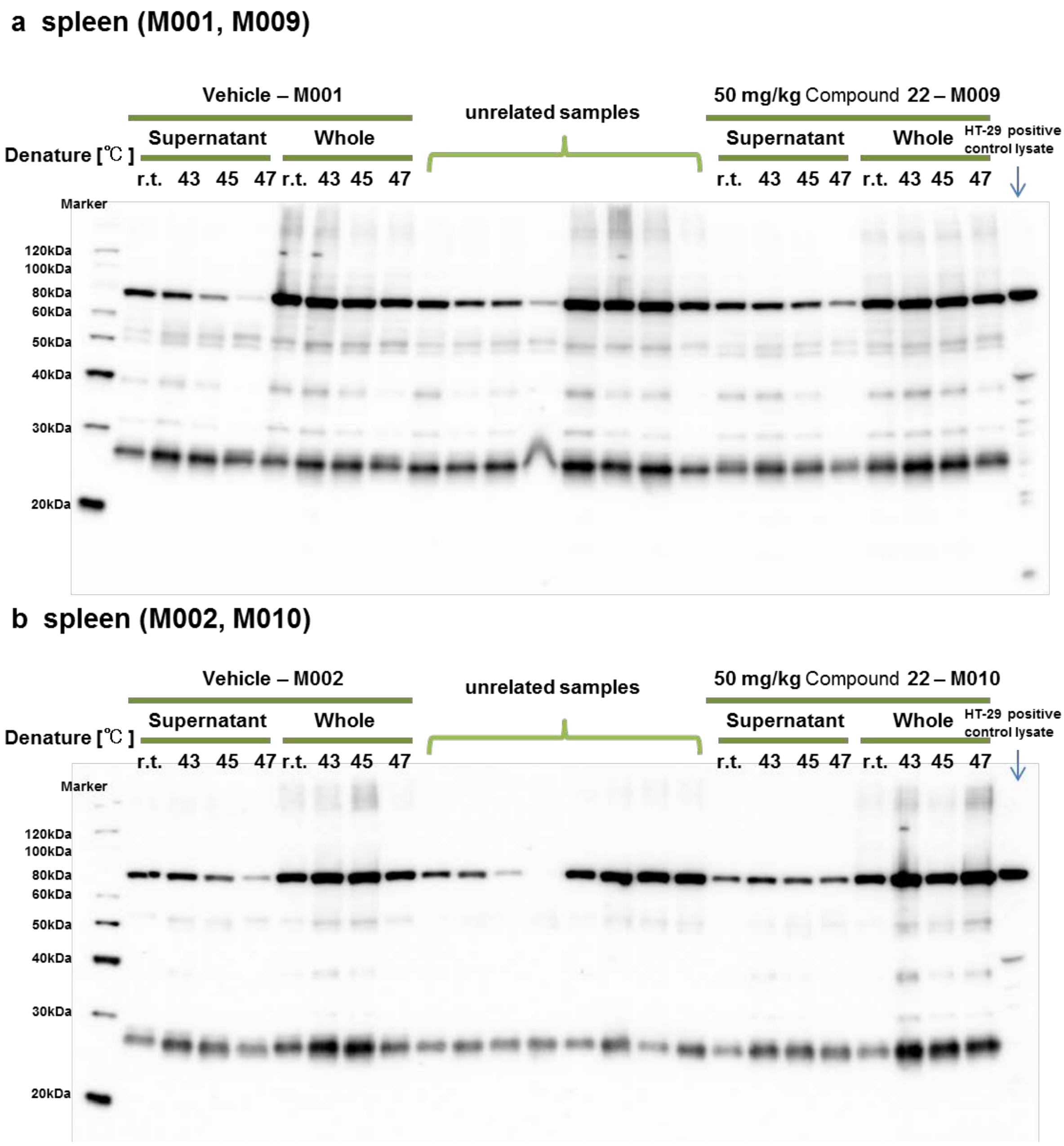

Supplementary Figure 11. All full-length Western blotting images in Figure 7

c spleen (M003, M011)

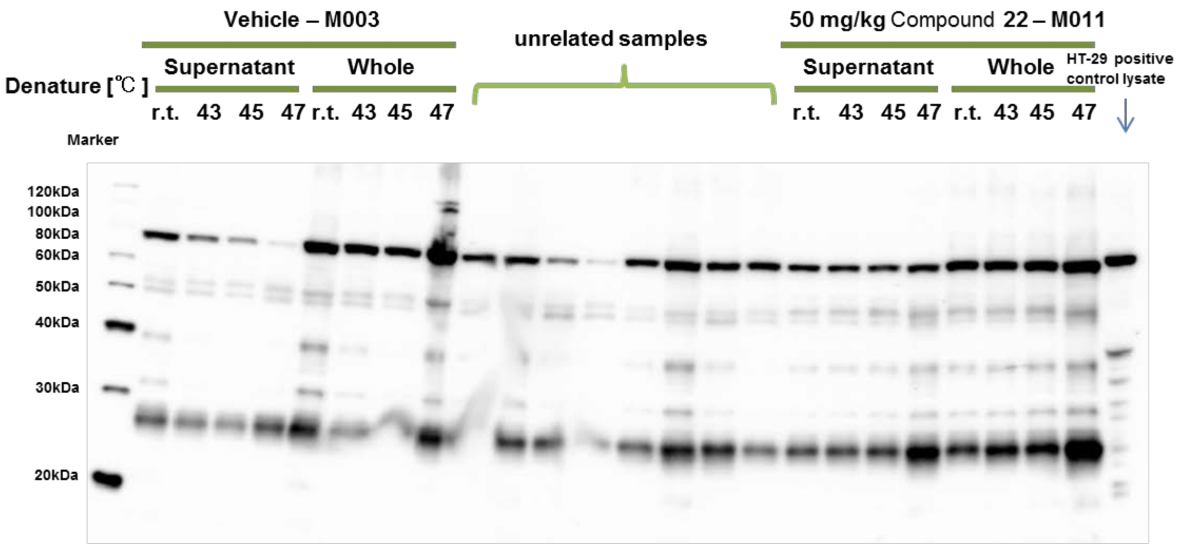

d spleen (M004, M012)

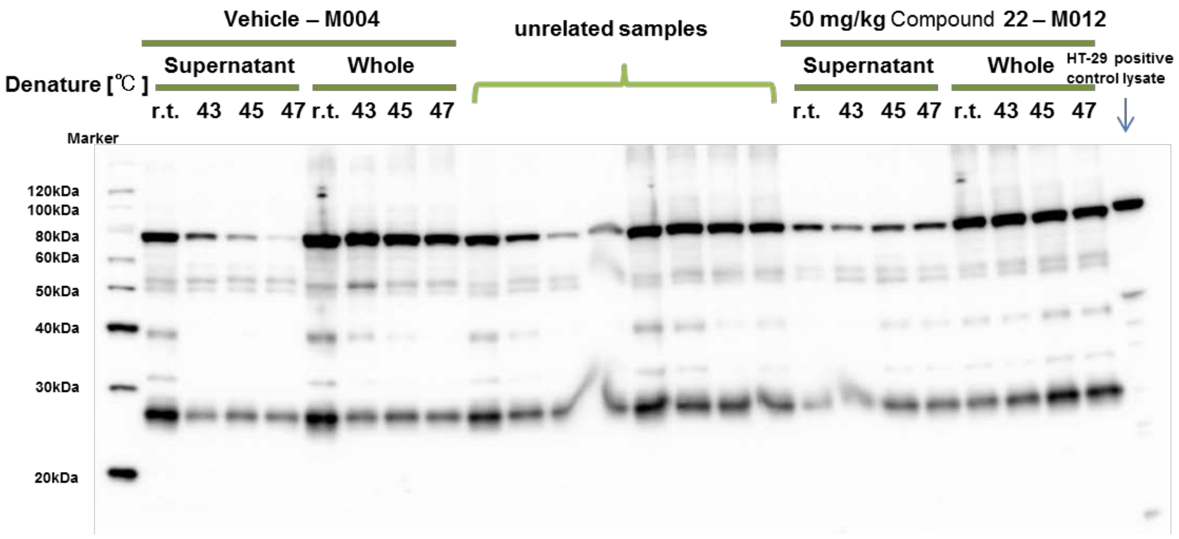

Supplementary Figure 11. All full-length Western blotting images in Figure 7 (continued)

e brain (M001, M009)

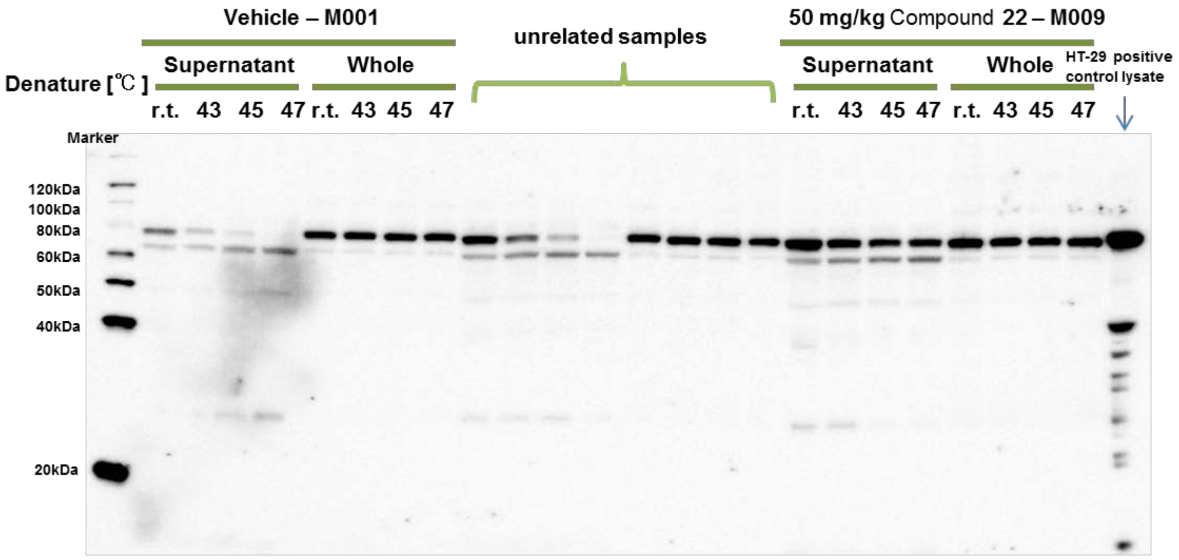

f brain (M002, M010)

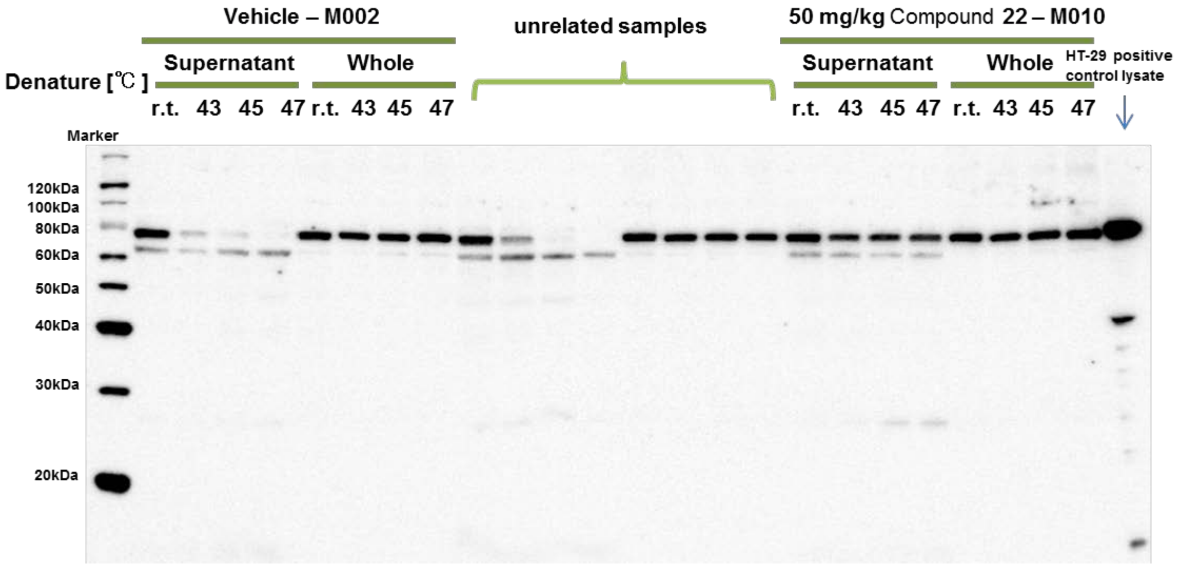

Supplementary Figure 11. All full-length Western blotting images in Figure 7 (continued)

g brain (M003, M011)

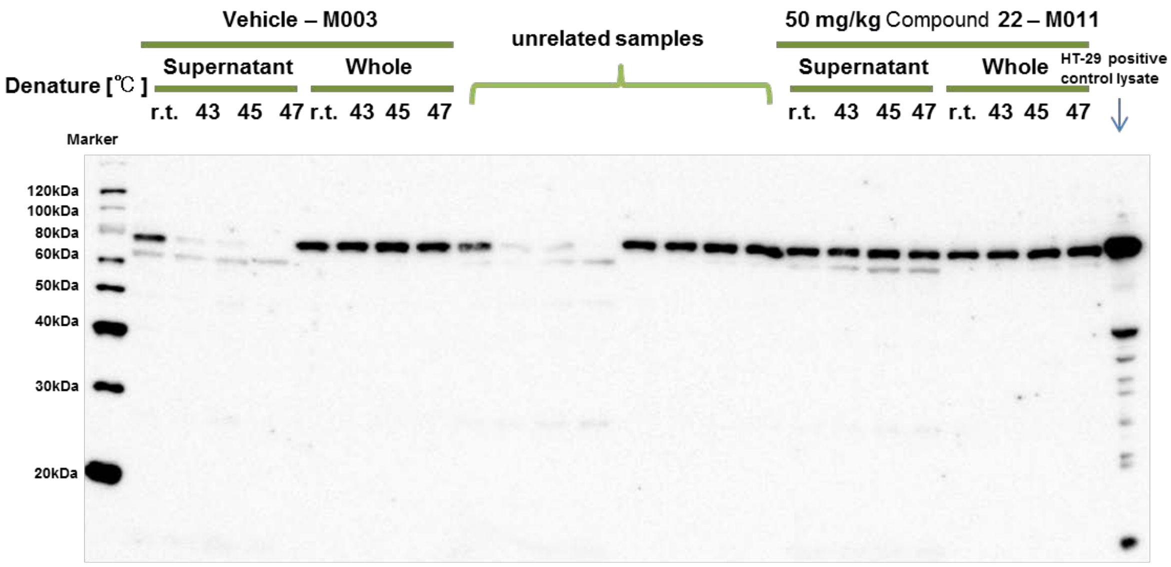

h brain (M004, M012)

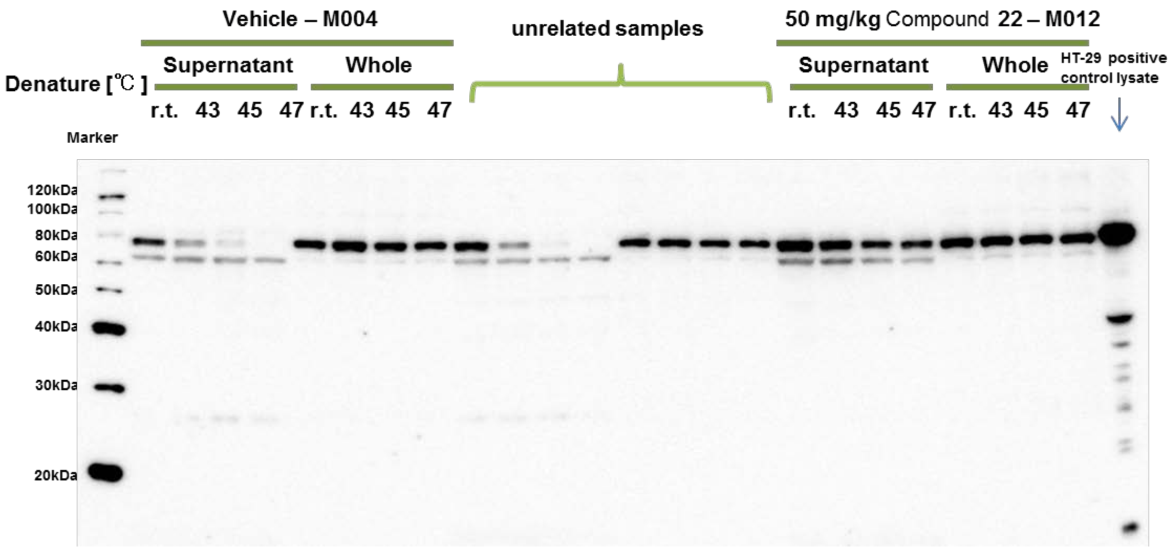

Supplementary Figure 11. All full-length Western blotting images in Figure 7 (continued)

Full images of figure 7a are shown in (a) spleen (M001, M009), (b) spleen (M002, M010), (c) spleen (M003, M011), and (d) spleen (M004, M012). Full images of figure 7b are shown in (e) brain (M001, M009), (f) brain (M002, M010), (g) brain (M003, M011), and (h) brain (M004, M012).Total cell extracts from HT-29 cells serve as a positive control.

Supplementary Table 1. Inhibitory activity

Compound ID	Chemical Structure	hRIPK1 enzyme (Ki)	mRIPK1 enzyme (Ki)	HT29-Necroptosis (IC50)	human_ITDRF-CETSA (EC50)	mouse_ITDRF-CETSA (EC50)
12	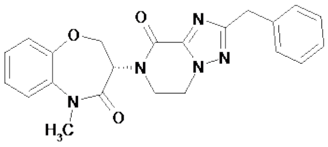	72<br>(58 - 90)	> 3,300	170<br>(120 - 230)	92<br>(47 - 180)	
14	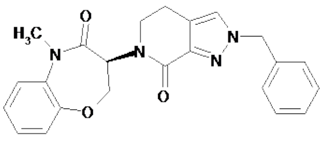	5.0<br>(4.0 - 6.2)	1,400<br>(1,200-1,700)	18<br>(14 - 23)	1.9<br>(0.5 - 7.7)	
15	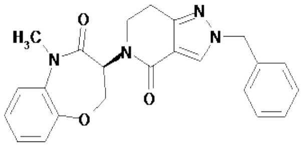	410<br>(320 - 530)	> 3300	1,700<br>(1,000-2,700)	4,700<br>(330-66,000)	> 30,000
16f	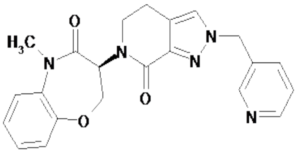	41<br>(34 - 50)	> 3,300	150<br>(63 - 370)	290<br>(140 - 610)	
16h	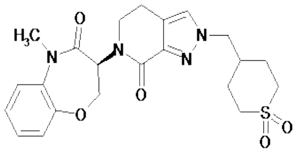	650	> 3,300	10000	6,900<br>(180-260,000)	
16p	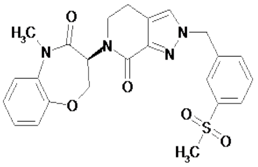	1,100<br>(870-1,500)	> 3,300	4,500<br>(3,700-5,300)	4,000<br>(820-20,000)	
16q	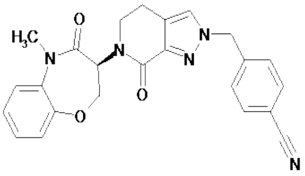	58<br>(42 - 80)	> 3,300	200<br>(120 - 360)	110<br>(80 - 150)	
18	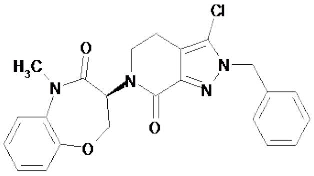	0.64<br>(0.60-0.68)	98<br>(78 - 120)	1.5<br>(1.2 - 1.9)	2.5	
22	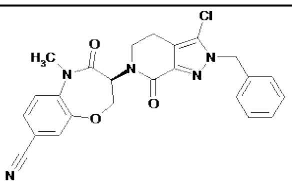	1.5<br>(1.3 - 1.6)	140<br>(120-160)	2.0<br>(1.5 - 2.7)	6.5<br>(4.3 - 9.8)	120<br>(91 - 160)

Supplementary Table 1. Inhibitory activity (continued)

Compound ID	Chemical Structure	hRIPK1 enzyme (Ki)	mRIPK1 enzyme (Ki)	HT29-Necroptosis (IC50)	human_ITDRF-CETSA (EC50)	mouse_ITDRF-CETSA (EC50)
23	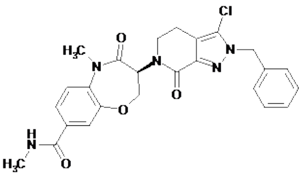	1.5 (1.4 - 1.6)	130 (110 - 150)	1.8 (0.15-21.0)	2.7 (1.3 - 5.3)	290 (250 - 340)
25	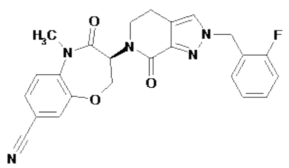	4.1 (3.8 - 4.4)	840 (720 - 970)	8.2 (6.7 - 9.9)	4.9 (1.0 - 24)	1,600 (1,200 - 2,100)
A	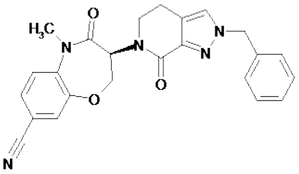	5.8 (5.1 - 6.6)	2,300 (2,100-2,600)	24 (15 - 39)	4.0 (1.1 - 14)	
GSK-compound 27	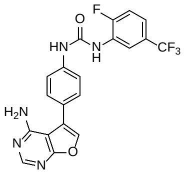	2.7 (2.5 - 3.0)	3.0 (2.7 - 3.4)	300 (160 - 580)	1,100 (700-1,700)	1,600 (1,200 - 2,200)
Nec-1	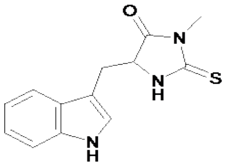	2,200 (1,600-3,000)	> 3300	610 (470-780)	1,100 (500-2,400)	2,800 (2,200 - 3,600)

Necrostatin-1 (Nec-1) was purchased from Sigma (M6006). Synthesis procedures for the other chemical inhibitors except for compound **A** were described in a previous article[2]. Compound **A** was synthesised in a manner similar to that described for the synthesis of all other compounds[2].

LC-MS (ESI) m/z 428.2 [M + H]+

1H NMR (300 MHz, CDCl3) δ 2.72 (1H, dt, J = 15.6, 4.7 Hz), 3.00-3.16 (1H, m), 3.39 (3H, s), 3.47-3.61 (1H, m), 4.12-4.24 (1H, m), 4.45 (1H, dd, J = 10.0, 8.1 Hz), 4.69 (1H, dd, J = 11.9, 10.0 Hz), 5.35 (2H, s), 5.94 (1H, dd, J = 11.9, 8.1 Hz), 7.15 (1H, s), 7.21-7.40 (6H, m), 7.47 (1H, d, J = 1.9 Hz), 7.56 (1H, dd, J = 8.3, 1.9 Hz).

CETSA, cellular thermal shift assay; EC_{50}, half-maximal effective concentration; IC_{50}, half-maximal inhibitory concentration; ITDRF, isothermal dose-response fingerprint; RIPK1, receptor interacting protein 1 kinase.

Supplementary Table 2. Unbound fraction in plasma and brain

	Compound **22**	Compound **25**
Plasma	0.060	0.130
Brain	0.022	0.067

Every experiment: N=3 except for brain compound **22** (N=2).

Supplementary Table 3. Plasma, liver, and brain concentrations of compound 22 at 1 h after oral administration to C57BL/6J mice

Dose, 50 mg kg^{-1}	Concentration (μg ml^{-1} or μg g^{-1})
Plasma	2.031 ± 0.427
Spleen	5.132 ± 1.582
Brain	6.589 ± 2.516

Mean ± s.d. N=4.

Reference

1. Cheng, Y. & Prusoff, W. H. Relationship between the inhibition constant (K1) and the concentration of inhibitor which causes 50 per cent inhibition (I50) of an enzymatic reaction. *Biochem. Pharmacol.* **22**, 3099-3108 (1973).

2. Yoshikawa, M. Discovery of 7-Oxo-2,4,5,7-tetrahydro-6H-pyrazolo[3,4-c]pyridine Derivatives as Potent, orally available, and Brain-penetrant Receptor Interactiong Protein 1 (RIP1) Kinase Inhitors Guided by Structure Kinetics Relationship. (Under submission).
